# Screening ASb_2_O_6_ (A = Mg, Ca, Sr, Ba, or Cd) for High-Performance Transparent Conducting Oxides

**DOI:** 10.1021/acs.chemmater.6c00854

**Published:** 2026-06-12

**Authors:** Romain Claes, Ke Li, Fatima Sajid, Alexander G. Squires, Robert G. Palgrave, David O. Scanlon

**Affiliations:** † School of Chemistry, 83415 University of Birmingham, Edgbaston, Birmingham B15 2TT, U.K.; ‡ Department of Chemistry, 4919 University College London, 20 Gordon Street, London WC1H 0AJ, U.K.

## Abstract

Transparent conducting oxides (TCOs) play an indispensable role in modern optoelectronic technologies. However, industry primarily relies on a limited range of *n*-type oxides, severely constraining the design flexibility and optimization of optoelectronic device architectures. Expanding the range of known TCOs would significantly broaden the available electron affinities, facilitating precise energetic matching and thereby enhancing device performance. Recently, Sb­(V)-based oxides have emerged as promising candidates, introducing an unexplored class of potential TCOs. In this study, we performed a computational screening of the ASb_2_O_6_ series (A = Mg, Ca, Sr, Ba, or Cd) to identify promising TCO candidates. Our analysis reveals that Ga-doped MgSb_2_O_6_ has a moderate carrier concentration of around 10^17^ cm^–3^, which can be further enhanced with higher annealing temperatures, while Y-doped CdSb_2_O_6_ is predicted to be a degenerate TCO. Experimental characterization of solid-state synthesized Y-doped CdSb_2_O_6_ powders corroborates these computational predictions, where Y incorporation leads to a Burstein–Moss widening of the optical band gap and a 0.1 eV shift of XPS core levels to higher binding energies, confirming a Fermi-level rise due to *n*-type doping. In addition, the resonant nature of Y_Cd_ preserves high mobility and reinforces the material’s strong potential for practical applications in next-generation optoelectronic devices.

## Graphical abstract

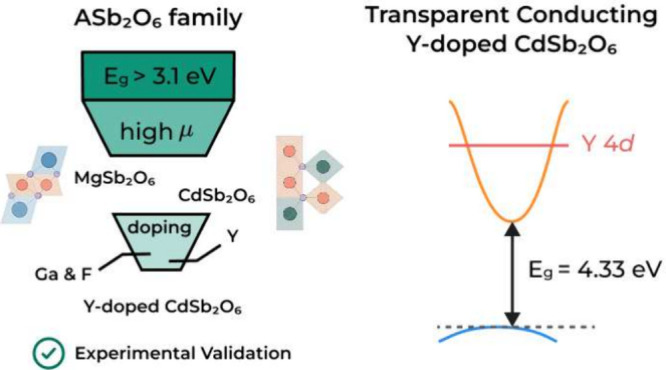

## Introduction

Antimonates have attracted attention over several decades for their notable photocatalytic properties, primarily due to their wide band gaps and chemical stability. These characteristics have enabled their use in photodegradation processes,^1–4^ as well as in water splitting^5,6^ and gas sensing applications.^7–11^ Additionally, these materials have been also studied for their photoluminescence properties, both in undoped form and when doped with ions such as Bi^3+^, La^3+^, or Eu^3+^.^12–15^ However, ASb_2_O_6_ compounds remain relatively underexplored in the context of TCOs, despite early investigations into their structural and electronic properties.^16,17^ More recently, a resurgence of interest in Sb­(V)-based oxides has led to the identification of ZnSb_2_O_6_ and Sb_2_O_5_ as promising candidates for next-generation *n*-type TCOs.^18–20^ In fact, these materials present several desirable properties, including wide optical band gaps, deep band edges, favorable band alignments for device integration, and degenerate *n*-type conductivity when doped with suitable elements such as gallium (Ga) or fluorine (F) (for ZnSb_2_O_6_ and Sb_2_O_5_, respectively). In these oxides, Sb­(V) adopts the classic (*n*–1)*d* ^10^ *ns* ^0^ *np* ^0^ electronic configuration – shared by post-transition cations in state-of-the-art TCOs – but also a specific arrangement of corner- and edge-sharing SbO_6_ octahedra. Together, these features often provide the highly dispersed conduction bands, which are essential for achieving high electron mobility in *n*-type TCOs.

Building on the promising results of these two systems, we extended our exploration to ASb_2_O_6_ compounds beyond ZnSb_2_O_6_ (A = Mg, Ca, Sr, Ba, Cd) in search of alternative candidates for practical TCO applications. In this work, we first examined the electronic structure of these materials, where wide band gaps were found going from 4 eV in the case of MgSb_2_O_6_ to very large values of more than 5.5 eV in Ca, Sr and BaSb_2_O_6_. The transport properties of the different Sb­(V) oxides were also investigated using a combination of the exact solution of the Boltzmann transport equation (BTE) and a model-based approach for the impurity scattering. Our findings indicate that MgSb_2_O_6_ and CdSb_2_O_6_ exhibit excellent transport properties at both low and high carrier concentration regimes, positioning them alongside other state-of-the-art oxides. Our work also reveals a strong correlation between the atomic packing factor (a measure of how efficiently atoms are packed in a crystal structure) and carrier mobility, offering a potential approach for rapid screening of promising candidates within this class of materials. Finally, the defect chemistry of the two most promising candidates – MgSb_2_O_6_ and CdSb_2_O_6_ – was investigated. The undoped MgSb_2_O_6_ is highly insulating, with a self-consistent Fermi level positioned deep within the band gap. Gallium and fluorine doping significantly enhance the electron concentrations in MgSb_2_O_6_, which can be further optimized by tailoring annealing temperatures and temperature-dependent band gap modulation. In contrast, intrinsic defect calculations for CdSb_2_O_6_ reveal a wide *n*-type doping window of around 2.05 eV, enabling effective yttrium doping. Y_Cd_ behaves as a resonant dopant species with low formation energy, enhancing conductivity without perturbing the conduction band minimum (CBM).^21–23^ The self-consistent Fermi level is located within the CBM, yielding degenerate *n*-type TCO behavior in Y-doped CdSb_2_O_6_. Experimental validation on Y-doped CdSb_2_O_6_ showed a Burstein–Moss widening of the optical band gap, and the shifts to higher binding energies in XPS measurements confirm the increase in the Fermi level during *n*-type doping.

## Computational Methodology

All the DFT calculations in this work were performed with the Vienna *ab initio* simulation package (Vasp) code,^24,25^ with the exception of the phonon and transport calculations of the iterative solution of the BTE where the software Abinit was used.^26–28^ Properties based on the density functional perturbation theory (DFPT) – e.g., phonon dispersions, iterative BTE (IBTE) and ionic dielectric constant calculations – were performed using the Perdew–Burke–Ernzerhof for solids (PBEsol)^29^ exchange-correlation (XC) functional within the generalized gradient approximation (GGA). The PBE0 hybrid functional^30^ was used for the remaining calculations, as it has been shown to accurately predict the electronic structure of Sb­(V) oxides and wide band gap oxides in general. Plane-wave cutoff and **k**-point sampling were converged for both Vasp and Abinit, resulting in a cutoff energy of 450 eV and *k*-point grids of 4 × 4 × 2 (MgSb_2_O_6_), 4 × 4 × 5 (CaSb_2_O_6_), 4 × 4 × 3 (SrSb_2_O_6_ and BaSb_2_O_6_), 5 × 5 × 5 (CdSb_2_O_6_) for Vasp calculations. For the Abinit calculations, an additional constraint was applied, requiring a minimum density of approximately 1500 points per reciprocal atom, which typically ensures converged phonons within DFPT.^31^ This resulted in **k**/**q**-point grids of 5 × 5 × 3 (MgSb_2_O_6_), 5 × 5 × 6 (CaSb_2_O_6_ and CdSb_2_O_6_), 6 × 6 × 5 (SrSb_2_O_6_ and BaSb_2_O_6_) and an energy cutoff of 45 Ha was used for all the calculations. The band alignments were computed using the PBE0 functional and the core and vacuum energies from a 30 Å thick (001) surface slab with 30 Å of vacuum using the Surfaxe package.^32^ Band structures and densities of states were calculated using the Sumo package.^33^

The charge transport calculations were performed using both Amset and Abinit, using a similar methodology as in refs. 20,34 The Abinit code allows us to compute the IBTE, without relying on common approximations that often lead to significant discrepancies with the exact solution of the BTE.^35,36^ Convergence in the IBTE mobility is achieved when three successive grids are within a 5% range. The converged mobility were obtained using *k*/*q*-meshes of 120 × 120 × 72 (MgSb_2_O_6_), 110 × 110 × 132 (CaSb_2_O_6_), 120 × 120 × 100 (SrSb_2_O_6_), 120 × 120 × 144 (CdSb_2_O_6_) and 108 × 108 × 90 (BaSb_2_O_6_). The convergence studies can be seen in the Supporting Information (Figure S1). The inclusion of dynamical quadrupoles (Q*)^37,38^ results in a negligible change in mobility (less than 1%) for CdSb_2_O_6_ and MgSb_2_O_6_ (see Table S1), and was therefore omitted for the rest of the study. On the other hand, Amset was mainly used for the impurity-limited mobility (IMP) in this work but the other phonon related mechanisms – acoustic deformation potential scattering (ADP), and polar-optical scattering (POP) – were also computed for comparison (see Figure S2). The input parameters for the Amset calculations can also be seen in the Supporting Information.

The supercell approach was used for defect calculations to reduce the interactions between periodic defects, where 90-atom supercells for MgSb_2_O_6_ and CdSb_2_O_6_ were generated from their corresponding primitive cells using the doped Python package.^39^ The ShakeNBreak package was used to generate 10 different distorted structures for each defect, where the defective supercells perform Γ-point-only relaxations to identify the ground and metastable defect structures.^40–42^ The identified ground-state structure was further optimized with a converged Γ-centered 2 × 2 × 2 **k**-point mesh to obtain its total energy, which can be incorporated into the equation below to calculate the formation energy of a defect^43^

1 $$\Delta E_{form}^{\left(D,q\right)}=\left(E^{\left(D,q\right)}-E^{H}\right)+\underset{i}{\sum }n_{i}\left(E_{i}+\mu_{i}\right)+q\left(E_{F}+\epsilon_{vbm}\right)+E^{corr}$$

where the first term accounts for the energy difference between the pristine supercell (*E* ^*H*^) and the defective supercell with the charge state *q* (*E* ^(*D,q*)^), the second part indicates the change in Gibbs free energy when *n* atoms of species *i* with its elemental reference energy *E*_*i*_ is added to or removed from its bulk supercell (μ_*i*_ is the formal chemical potential), the third term is the energy cost associated with adding or removing charge *q* to the defect site from the system (*E*_*F*_ is the Fermi energy and ϵ_*vbm*_ is the eigenvalue of the host valence band maximum), the final term (*E* ^*corr*^) is the correction term which accounts for any spurious interactions introduced by the finite-size supercell approximation. The doped Python package was used to analyze all defect calculations, and the py-sc-Fermi package was applied for self-consistent Fermi level predictions.^44^ The unfolded band structure for the defective supercell was carried out using the easyunfold package.^45^ Defect calculations followed the recently proposed guidelines for reproducibility^46^ and the data will be available upon publication on Zenodo (doi.org/10.5281/zenodo.17287761).

## Experimental Methodology

CdSb_2_O_6_ was synthesized by adapting the method of Castro et al.^47^ CdO and Sb_2_O_3_ were ground together and heated in air at 1000 °C for a total of 48 h in an alumina crucible, with two intermediate regrinding steps. A 15% excess of Sb_2_O_3_ was used to account for volatilisation. Doped CdSb_2_O_6_ was produced by adding Y_2_O_3_ to the reaction mixture in the Cd: Y ratio 10:1 (a 9.1% assuming the Y only replaces the Cd), otherwise the synthesis was identical.

Powder X-ray diffraction (PXRD) was carried out in foil transmission geometry using a Stoe Stadi-P X-ray diffractometer with a Cu Kα_1_ radiation source (λ = 1.5406 Å, 40 kV, 30 mA). Data were collected from 5 to 50° at 0.5° per step for 15 s.

X-ray photoelectron spectroscopy (XPS) was performed using a Thermo Scientific Kα spectrometer, which uses a monochromatized Al Kα X-ray source (*hν* = 1486.6 eV), a hemispherical analyzer, and a two-dimensional detector. The electron energy analyzer consists of a double focusing 180° hemisphere with a mean radius of 125 mm, operated in constant analyzer energy (CAE) mode, and a 128-channel position sensitive detector. Measurements were conducted with a 400 μm spot size using a dual-beam flood gun (electron and Ar^+^ ion) with a 100 mA current. The pass energy was 200 eV for the survey spectra and 40 eV for the core levels. The Thermo Avantage v5.9925 software package was used for XPS spectral analysis, and all spectral fittings were performed with a Shirley background. UV–vis spectra were recorded in diffuse reflectance mode using a PerkinElmer Lambda-950 spectrometer. Samples were ground and fixed to carbon tape on a glass slide. Diffuse reflectance spectra were recorded from 200 to 2000 nm. The reflecting reference used was a barium sulfate pellet. Reflectivity (R) data were converted using the Kubelka–Munk relationship: F­(R) = (1 – R)­2/2R.

## Crystal Structure

As shown in Figure 1, the Sb­(V) oxides studied in this work exhibit two different crystal structures: MgSb_2_O_6_ adopts the trirutile framework of ZnSb_2_O_6_, while the other ASb_2_O_6_ materials studied in this work crystallize in a distinct rosiaite PbSb_2_O_6_-type structure. This structural change is driven by the increase of the A-site cation radius that destabilizes the rutile structure in favor of the rosiaite structure type. In both structures, the oxygen presents a similar coordination environment which lead to an infinite network of edge-sharing SbO_6_ octahedra. However, while the rosiaite structure exhibits only 2D layers (“sheets”) of edge-sharing octahedra, the trirutile structure displays corner-sharing octahedra in addition to the edge-sharing chains, as shown in Figure 1. In the rosiaite structures, the increasing radius of the A-cations, going from Cd to Ba, also directly affects the O–A distances (see Table S2), leading to a reduction in the covalent character of the bonding. This is confirmed by the Crystal Orbital Bond Index (COBI) approach,^48^ which quantifies the degree of covalency in specific bonds. The COBI values decrease from 0.11 for Cd to 0.072 for Ca, 0.063 for Sr, and 0.049 for Ba, closely correlating with the increasing O–A bond distances (see Table S2). Overall, our calculated lattice parameters are in excellent agreement with the experimental results as seen in Table 1.

**Figure 1:**
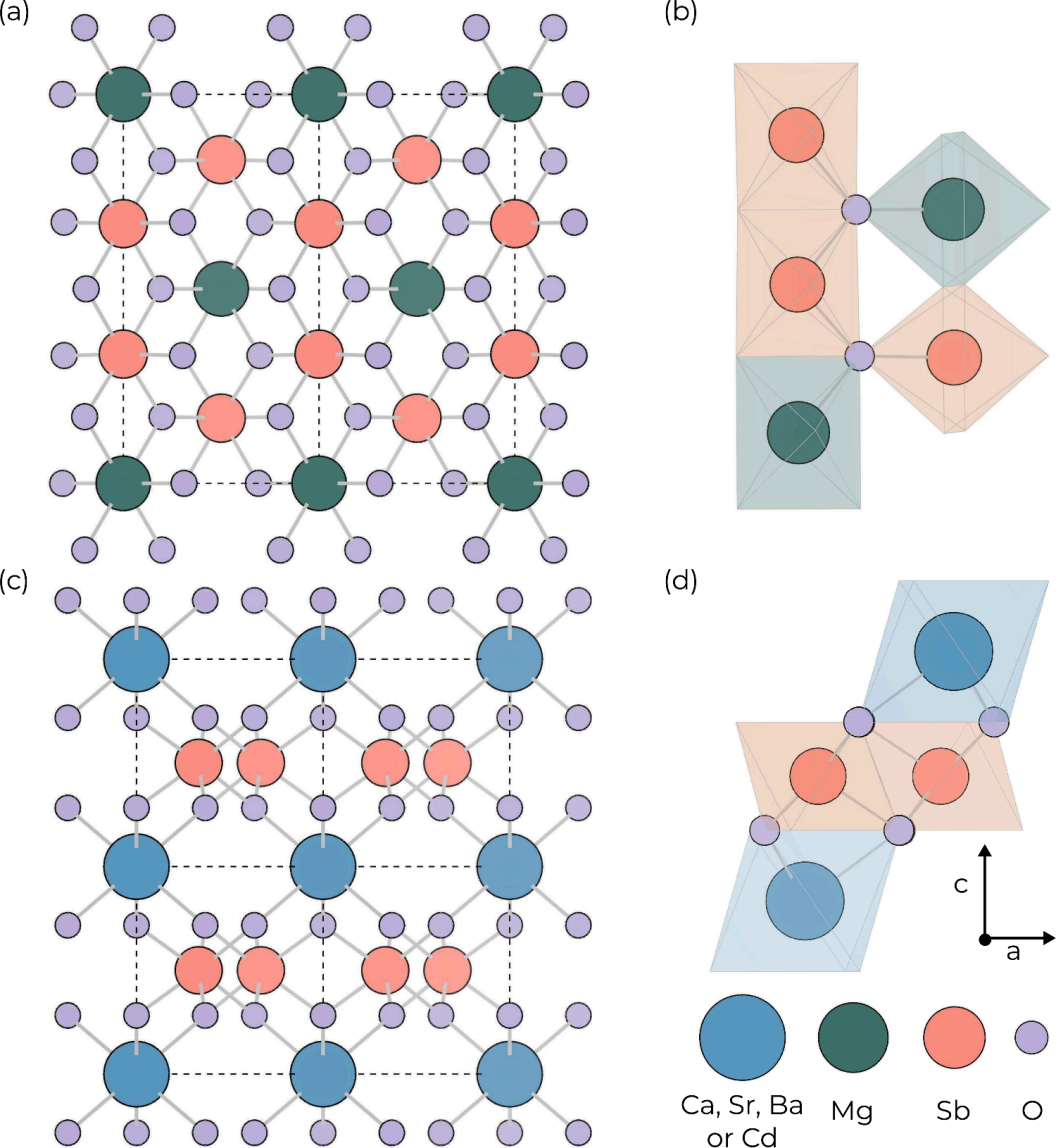
(a) Crystal structure of MgSb_2_O_6_ (trirutile structure) with (b) the corresponding octahedral connectivity displaying a combination of corner- and edge-sharing SbO_6_ octahedra. (c) Crystal structure of (Ca, Sr, Ba, or Cd)­Sb_2_O_6_ (rosiaite structure) with (d) the corresponding edge-shared octahedral connectivity.

**Table 1:** Structural Properties of the Different ASb_2_O_6_ Compounds Studied in This Work

		*a* (Å)		*c* (Å)	
material	space group	PBE0	Exp.	PBE0	Exp.
MgSb_2_O_6_	*P*4_2_/*mnm*	4.662	4.640–4.664 ^10,17,49,50^	9.244	9.229–9.261 ^10,17,49,50^
CaSb_2_O_6_	*P* 31*m*	5.249	5.241 ^51^	5.059	5.022 ^51^
SrSb_2_O_6_	*P* 31*m*	5.282	5.272 ^51^	5.408	5.366 ^51^
BaSb_2_O_6_	*P* 31*m*	5.317	5.304–5.309 ^15,51^	5.827	5.760–5.763 ^15,51^
CdSb_2_O_6_	*P* 31*m*	5.251	5.237–5.325 ^2,15,17^	4.866	4.694 ^2,15,17^

## Electronic Structure

For all the Sb­(V) oxides studied in this work, the band structure exhibits a curved CBM located at the Γ-point, as shown in Figure 2(a) and (c) for MgSb_2_O_6_ and CdSb_2_O_6_, respectively (the band structures of (Ca, Sr, Ba)­Sb_2_O_6_ can be seen in the Supporting Information, Figure S3). In CdSb_2_O_6_, Cd­(II) also directly contributes to the conduction band, as commonly observed for post-transition metal, which is not the case for Mg­(II) in the trirutile MgSb_2_O_6_, as shown in the density of states (DOS) in Figure 2(a). This difference is also evident in the charge density plots (Figure 2(b) and (d)), where the CBM charge density in MgSb_2_O_6_ is primarily localized around O and Sb atoms, with no contribution from Mg. In contrast, in CdSb_2_O_6_, the charge density clearly extends onto the Cd atoms, consistent with the presence of Cd *s* orbital character at the CBM in the DOS.

**Figure 2:**
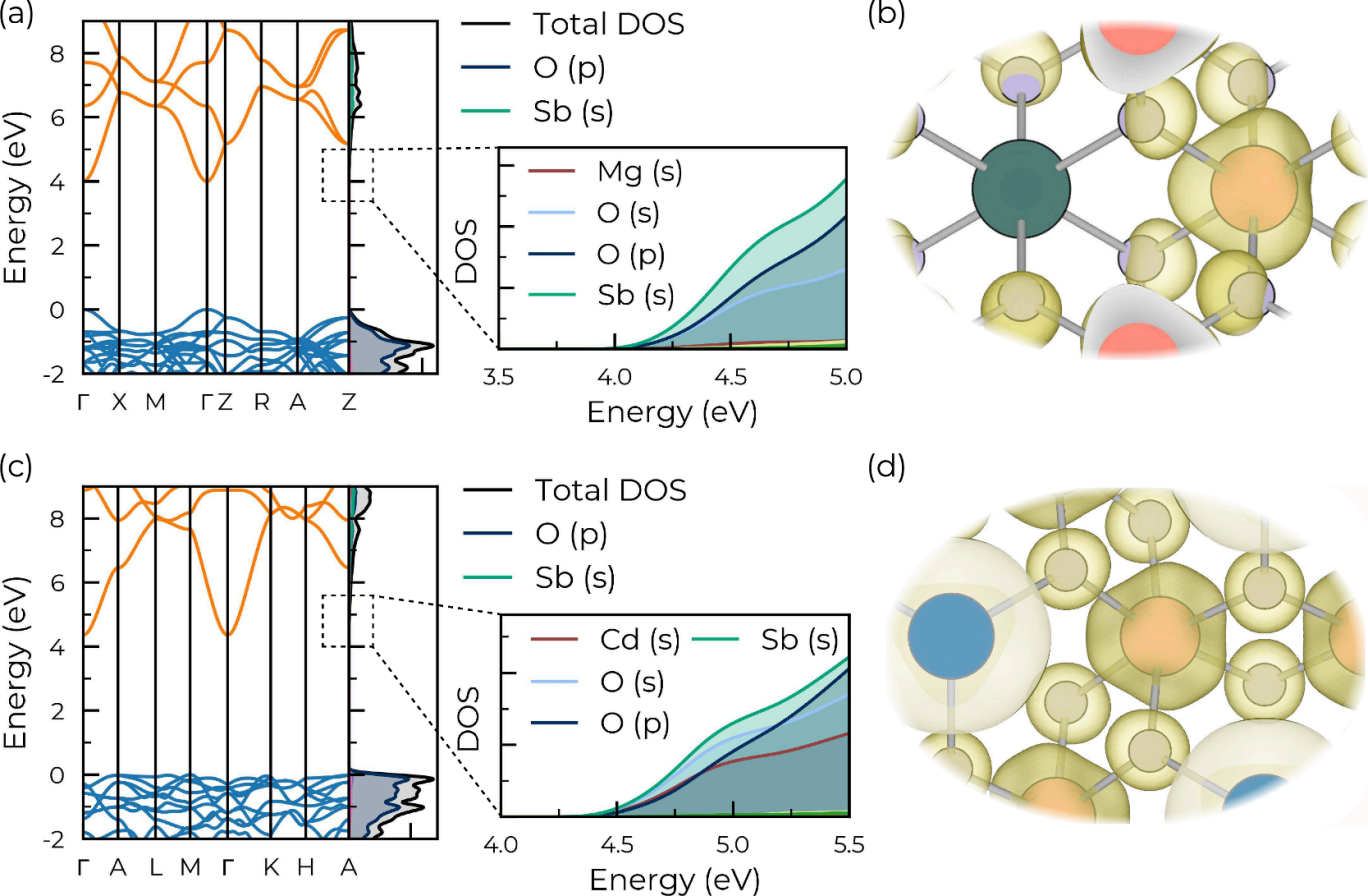
Electronic band structures of (a) MgSb_2_O_6_ and (c) CdSb_2_O_6_ computed using the PBE0 functional. The band structures of (Ca,Sr,Ba)­Sb_2_O_6_ are available in the Supporting Information. Corresponding charge density at the conduction band minimum for (b) MgSb_2_O_6_ and (d) CdSb_2_O_6_.

Figure 3 shows the absorption coefficient of the different oxides, derived from the real and imaginary parts of the complex dielectric function calculated via DFT. All the antimony oxides show good promise for transparency with fundamental band gaps that range from 4.0 eV in MgSb_2_O_6_ to around 5.6 eV in Ca, Sr, and BaSb_2_O_6_ as shown in Figure 3(a). In the case of trirutile MgSb_2_O_6_, the optical band gap is larger than the fundamental band gap because the first Γ – Γ transition is forbidden, as in ZnSb_2_O_6_,^18^ resulting in an estimated optical band gap of 4.2 eV close to experimental values.^10,17,49^ For the other antimony oxide, the comparison with experimental values is trickier due to the wide range of experimental measurements. The smaller band gap obtained in CdSb_2_O_6_ in comparison to the other rosiaite structure is directly linked to the Cd^2+^ contributing importantly to the CBM, as shown in the density-of-state (Figure 2(c)). The band gap results are summarized in Table 2.

**Table 2:** Band Gaps (indirect and optical estimated based on a Tauc plot) of the Different ASb_2_O_6_ Compounds Studied in This Work, Computed with the PBE0 Functional

	*E*_*g*_ (eV)		
material	indirect (PBE0)	estimated optical (PBE0)	Exp.
MgSb_2_O_6_	4.00	4.2	4.05 ^49^
			3.86 ^10^
			4.3 ^17^
CaSb_2_O_6_	5.59	5.54	3.59 ^5^
			3.9 ^52^
			5.0 ^17^
SrSb_2_O_6_	5.63	5.64	3.55 ^5^
			5.0 ^17^
BaSb_2_O_6_	5.62	5.64	3.46 ^5^
			3.34 ^15^
			>5.5 ^17^
CdSb_2_O_6_	4.33	4.32	2.95 ^15^
			>4.1 ^16^
			3.8 or 4.4 ^17^

**Figure 3:**
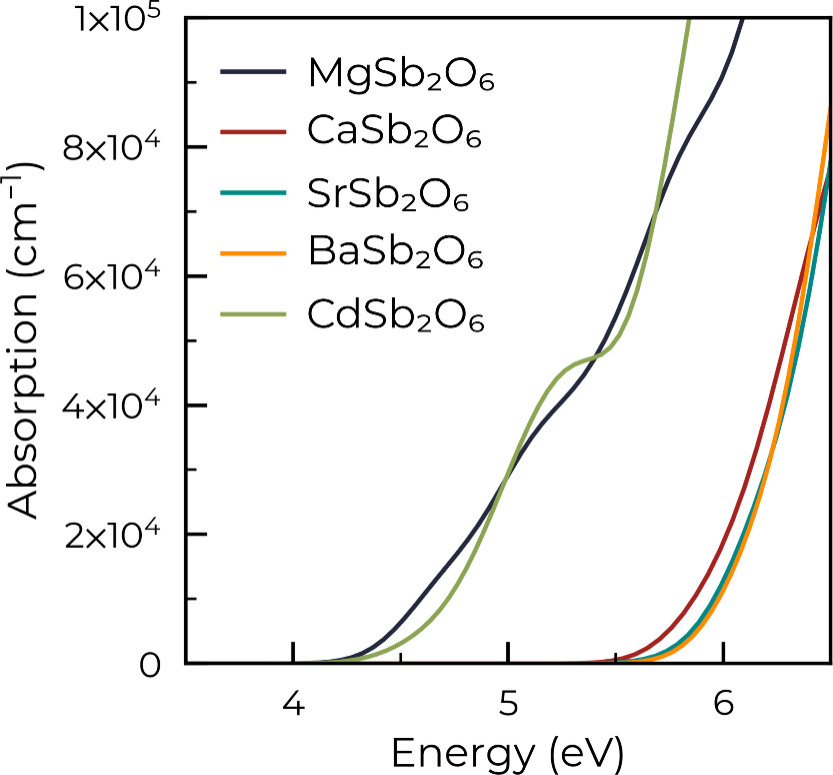
Band gap optical absorption spectra for the different Sb­(V) oxides.

**Figure 4:**
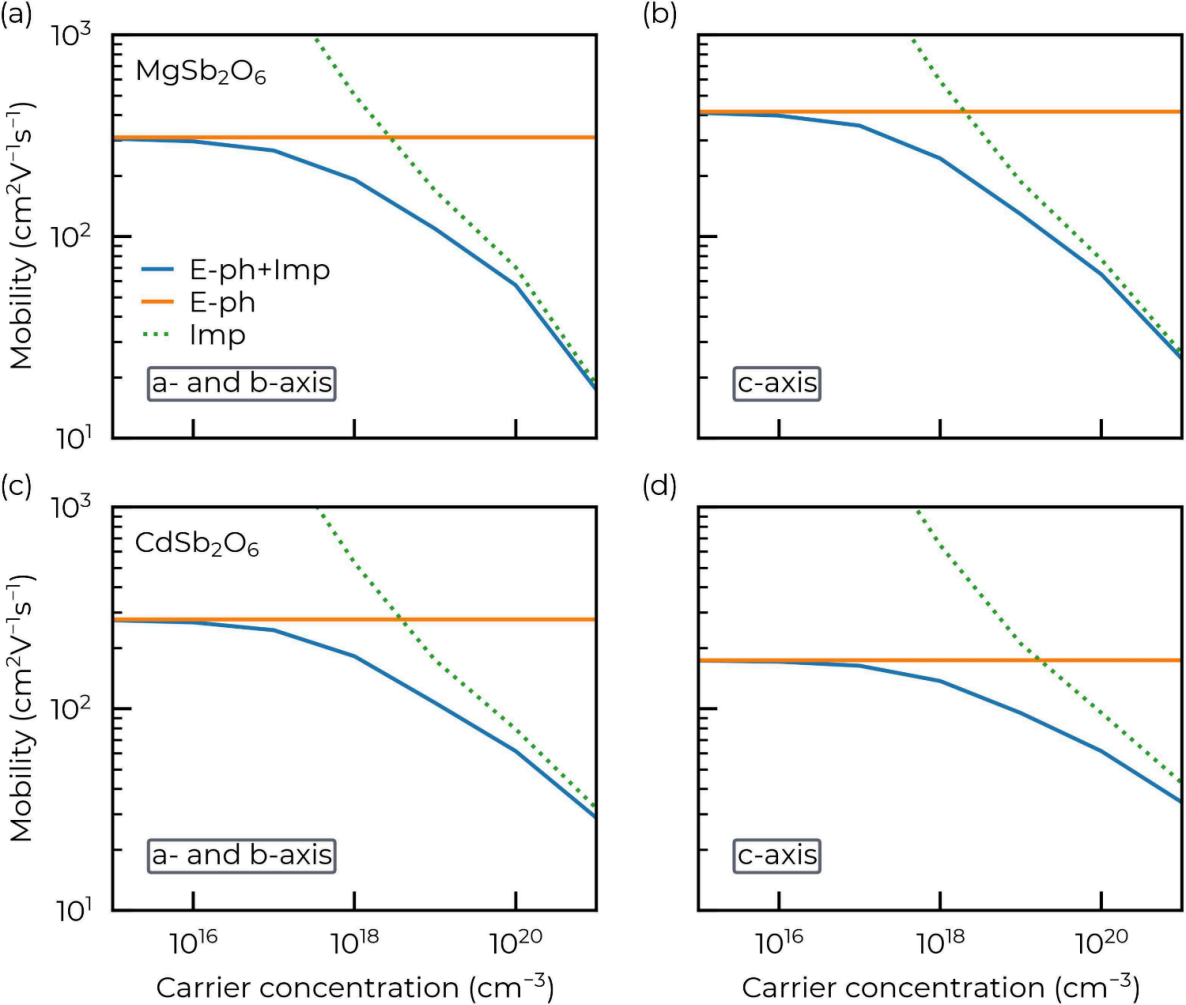
Computed mobilities of MgSb_2_O_6_ along the (a) *a*- and *b*-axes and (b) *c*-axis and CdSb_2_O_6_ along the (c) *a*- and *b*-axes and (d) *c*-axis. e-ph stands for electron–phonon (with this contribution computed using the iterative Boltzmann transport equation (IBTE) and Abinit), imp the impurity contribution (computed using a Brooks–Herring model as implemented in Amset), and e-ph+imp the sum of the two contributions using Matthiessen’s rule. The mobility results for (Ca, Sr, Ba)­Sb_2_O_6_ are available in the Supporting Information.

## Electron Transport Properties

Table 3 presents the electron mobility results at low and high carrier concentration regimes. Interestingly, the electron mobility is higher along the SbO_6_ edge-sharing octahedra (corresponding to the *c*-axis in the case of MgSb_2_O_6_ and the *a* and *b*-axis for the other Sb­(V) oxides). In all oxides, the Sb–O bond lengths remain nearly similar, with a consistent degree of covalency (COBI = ∼ 0.55). For the rosiaite structures, the observed decrease in mobility down the group can again be attributed to variations in the covalency of the A–O bonds, which directly affect the hybridization at the CBM. Notably, the DOS in Figure 2(c) reveals a non-negligible contribution from the Cd *s* orbitals, further reinforcing the metallic character of the CBM. In the case of trirutile MgSb_2_O_6_, the COBI values for the Mg–O bonds are slightly lower than those for Cd, ranging from 0.088 to 0.098 depending on the specific bond. However, as shown in Figure 2(a), the density of states (DOS) does not indicate a significant contribution from Mg. The high electron mobility in MgSb_2_O_6_ is more likely linked to the favorable combination of edge- and corner-sharing SbO_6_ octahedra, such as in ZnSb_2_O_6_.^18^

**Table 3:** Mobility Results (electron-phonon + impurity) at Low and High Carrier Concentrations (*n*)

	electron–phonon + impurity mobility (cm^2^ V^–1^ s^–1^)			
	low *n* (×10^15^ cm^–3^)		high *n* (×10^21^ cm^–3^)	
material	*a*- and *b*-axes	*c*-axis	*a*- and *b*-axes	*c*-axis
MgSb_2_O_6_	309.7	416.3	17.0	24.1
CaSb_2_O_6_	122.3	76.1	19.3	11.5
SrSb_2_O_6_	89.0	53.4	17.4	10.0
BaSb_2_O_6_	66.3	41.1	17.0	8.7
CdSb_2_O_6_	277.7	174.3	37.1	27.2

A clear correlation is observed between the atomic packing factor, i.e., the fraction of volume in a crystal structure occupied by constituent atoms, and the average phonon-limited mobility, as shown in Figure 5. Overall, the more densely the atoms are packed within the structure, the higher the intrinsic mobility tends to be. While this trend is expected due to its direct relation to bond covalency, it appears to hold particularly well for the Sb­(V) oxides studied in this work. Moreover, it is consistent with previously reported phonon-limited results for other Sb­(V) oxides, such as Sb_2_O_5_ and ZnSb_2_O_6_,^20^ using the same computational methodology. Sb_2_O_5_ crystallizes in a stacked monoclinic structure with edge- and corner-sharing octahedra and exhibits the highest phonon-limited mobility computed to date among Sb­(V) oxides.^20^ For ternary systems, the packing factor trend closely follows the ionic radius of the alkaline-earth cation: Zn^2+^ possesses the smallest ionic radius, followed by Mg^2+^ and Cd^2+^, while Ca^2+^, Sr^2+^, and eBa^2+^ are significantly larger, resulting in a markedly lower packing factor and reduced mobility.^53^ Beyond the packing factor, the octahedral connectivity also plays a role: the rosiaite structure features exclusively edge-sharing octahedra, whereas Sb_2_O_5_ and the trirutile structure exhibit both edge- and corner-sharing connectivity, which is generally associated with higher carrier mobility.

**Figure 5:**
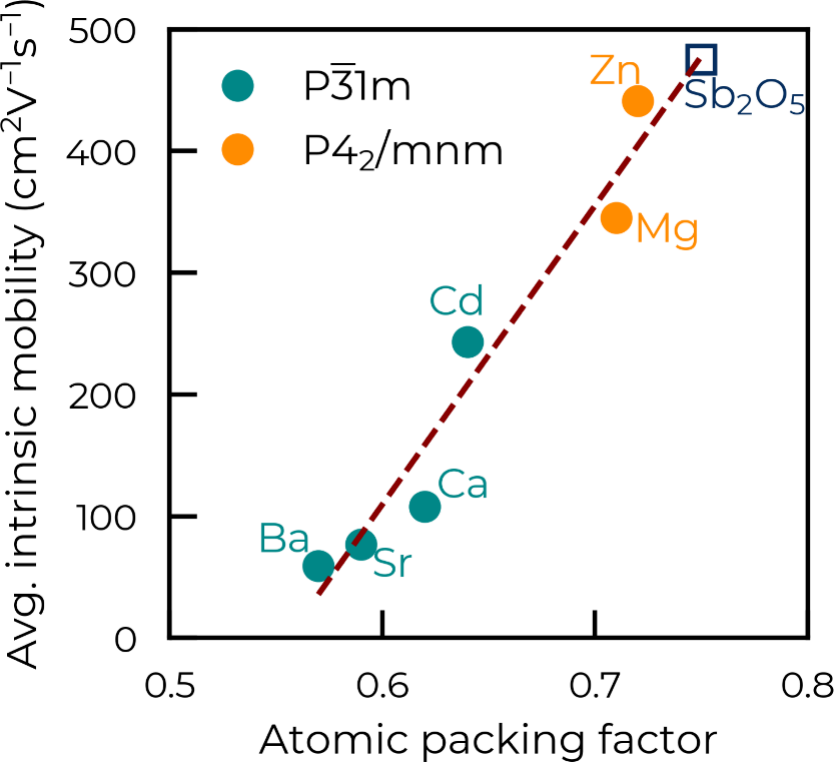
Average mobility with respect to the atomic packing factor. Mobility results for ZnSb_2_O_6_ and Sb_2_O_5_ can be found in ref 20.

Figure 4 presents the electron mobility results at different carrier concentrations for MgSb_2_O_6_ and CdSb_2_O_6_ computed with different scattering mechanisms included. As shown in Table 3, these two systems present the highest mobility results in this work in both low and high carrier concentration (*n*) regimes. The phonon-limited mobilities calculated iteratively using Abinit combined with the impurity-limited mobilities from Amset (e-ph+imp, blue line in Figure 4) represent our final results. The results for the other Sb­(V) oxides can be seen in Figure S2. The full Amset results as well as the mobilities decompose by mechanisms (POP, ADP and IMP). As observed in previous works,^20,54^ the phonon-limited mobility is largely underestimated when using Amset alone, and a more sophisticated formalism, such as the exact IBTE used in this work, is required to obtain more accurate phonon-limited mobilities.

As expected, phonon scattering dominates at low carrier concentration whereas the impurity scattering is the main mechanism at higher carrier concentration. In the high carrier concentration regime, an important difference can be seen in the impurity scattering between the two structure. While MgSb_2_O_6_ exhibits the highest mobility at low doping, its mobility decreases more sharply with increasing carrier concentration than in CdSb_2_O_6_. In contrast, in CdSb_2_O_6_ (and its Ca, Sr, and Ba analogs), impurity scattering is weaker, allowing the mobility to remain relatively high. To understand this difference, one must examine how impurity scattering is treated in the Amset model. The approach in Amset is based on the Brooks–Herring formalism, which incorporates input parameters such as the dielectric constant, effective masses, and screening length in addition to the spatial overlap between the wave function and the impurity potential, captured by the scattering matrix elements. Since these parameters possess values relatively similar for MgSb_2_O_6_ and CdSb_2_O_6_ (see Supporting Information), the observed difference in impurity scattering must stem from the matrix elements directly. This can again be explain by the orbital character of the CBM in both cases: in CdSb_2_O_6_, the CBM includes a significant contribution of the Cd *s* states, which is not the case of Mg *s* orbitals in MgSb_2_O_6_ (see the DOS in Figure 2(a) and (c) as well as the charge density for the CBM of MgSb_2_O_6_ in Figure 2(b)). As a result, in CdSb_2_O_6_, the wave function is spatially distributed across all atomic sites and the perturbation of the wave function due to the homogeneous impurity potential is relatively small, leading to weaker scattering. On the other hand, the impurity scattering matrix elements are larger in MgSb_2_O_6_ as the Coulomb impurity potential introduces a stronger perturbation.

## Intrinsic Defect Chemistry

With the most promising *n*-type mobility, MgSb_2_O_6_ and CdSb_2_O_6_ were chosen for further study. To achieve conductivity competitive with commercial TCOs, high carrier concentrations are also required. Carrier concentrations are determined via calculation of the self-consistent Fermi energy, which is set by defect formation. Defect formation energies are a function of the growth environment which are represented by the chemical potential term in eq 1. The bounds on reasonable chemical potentials are set by the stability limits of the host material with respect to competing phases. The thermodynamic stability regions of MgSb_2_O_6_ and CdSb_2_O_6_ were identified with respect to their competing phases, and are shown in Figure S4. The red dot indicates the most *n*-type (metal-rich/O-poor) conditions. The calculated formation energies of the competing phases and chemical potential limits are listed in Tables S3–S6.

The formation energies of the intrinsic point defects are shown on the transition level diagrams in Figures 6(a) and (b) for MgSb_2_O_6_ and CdSb_2_O_6_ under the most *n*-type (metal-rich/O-poor) conditions. For CdSb_2_O_6_, the considered intrinsic point defects consist of vacancies (*V*_O_, *V*_Sb_ and *V*_Cd_), interstitials (Cd_i_, Sb_i_, O_i_) and antisites (Cd_Sb_, Sb_Cd_ and Sb_O_). In MgSb_2_O_6_, the types of defects are analogous, with all Cd-related defects substituted by Mg. However, due to the difference in structure, MgSb_2_O_6_ has two crystallographically distinct oxygen sites labeled by their point symmetry, *C*_2*v*_ and *C*_*s*_. In both systems, the oxygen vacancies (*V*_O_) are low-energy donor defects, exhibiting negative-U behavior. This behavior was also seen in other Sb­(V) oxides and conventional TCOs.^19,55–57^ The (+2/0) transition level appears at 1.35 eV below the CBM in CdSb_2_O_6_, while in MgSb_2_O_6_, it is observed at 1.55 and 1.58 eV relative to the CBM for *V*_*O*_*C*2*v*__ and *V*_*O*_*Cs*__, respectively. The antisite defects Sb_Cd_ and Sb_Mg_ also show relatively low formation energies in their respective systems. The Sb_Cd_ defect has both a (+1/0) transition level in the conduction band and a relatively low formation energy making it a dominant contributor to the overall carrier concentration. Cation interstitials are shallow donors, but have high formation energies and therefore their effect on carrier concentration is negligible.

**Figure 6:**
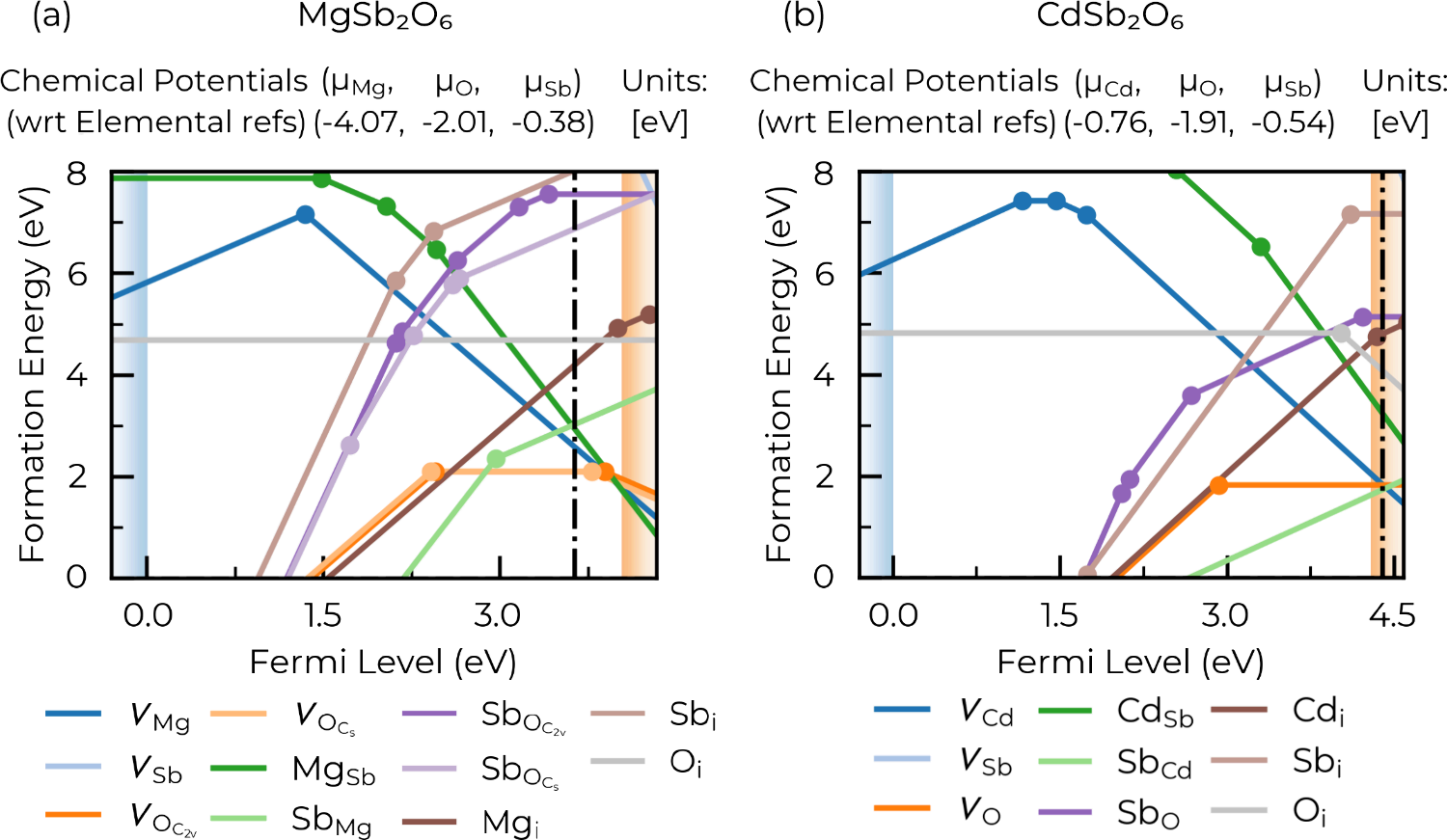
(a) Transition level diagram of intrinsic point defects in MgSb_2_O_6_ under metal-rich/O-poor (*n*-type) conditions, plotted using doped.^39^ The self-consistent Fermi level is located 0.40 eV below the CBM shown as the black dotted line.^39,44^ Each line corresponds to a specific defect in a given charge state, with the slope reflecting the charge state of the defect. The intersections between lines indicate thermodynamic charge transition levels. The shaded blue and orange regions denote the VBM and CBM, respectively. (b) Plot of the formation energy vs Fermi level of intrinsic point defects in CdSb_2_O_6_ under metal-rich/O-poor (*n*-type) conditions, with the same conventions as in panel a. The self-consistent Fermi level sits 0.10 eV above the CBM shown as the black dotted line.^39,44^

O_i_ in CdSb_2_O_6_ acts as an acceptor defect with (0/-2) transition occurs just below CBM. While O_i_ in MgSb_2_O_6_ stays in neutral charge state throughout all Fermi levels. Peroxide-like O–O dimers are formed in the two systems, where bond lengths of 1.43 Å are observed across all charge states in MgSb_2_O_6_ and a 1.45 Å O–O bond length is formed in the neutral state of CdSb_2_O_6_. The cation vacancies *V*_Cd_ and *V*_Mg_ behave as ultradeep acceptors. In CdSb_2_O_6_, *V*_Cd_^–2^ compensates donor defects, resulting in an *n*-type doping window of 2.05 eV.

The doping window is determined by the formation energy of the lowest-energy compensating defect at the corresponding band edge.^19^ Consequently, MgSb_2_O_6_ has a smaller doping window, as the Mg_Sb_^–3^ intersects the CBM at 1.74 eV. Notably, in MgSb_2_O_6_, *V*_Mg_ exhibits a 3-electron direct transition level of (+1/-2) that occurs at 1.34 eV above VBM. By looking at the relaxed defect structure of *V*_Mg_^+1^ in Figure S5­(a), a superoxide-like O–O dimer with a bond length of 1.30 Å was formed near the vacancy site, trapping three holes. This dimer formation was identified using the ShakeNBreak approach, resulting in an energy of 0.9 eV lower than the unperturbed defect structure relaxation (Figure S5­(c)). The unoccupied defect state sits close to the CBM, where the charge density is strongly localized on the oxygen dimer shown in Figure S5­(a) and (b). *V*_Mg_ in its fully ionized charge state (−2) then becomes stable as the Fermi level approaches the CBM.

The self-consistent Fermi levels, indicated by the black dotted lines in Figure 6, were predicted based on the typical annealing or synthesis temperatures -1200 K for CdSb_2_O_6_ and 1000 K for MgSb_2_O_6_, and then recalculated at room temperature (300 K).^9,10,15,17,44,58,59^ The overall *n*-type carrier concentration generated from the native point defects in CdSb_2_O_6_ is in between 10^17^ cm^–3^ and 10^18^ cm^–3^, which is approximately 4 orders of magnitude higher than that in MgSb_2_O_6_, as shown in Figures S6­(b) and S7­(b). This difference is also reflected in the self-consistent Fermi level positions: in CdSb_2_O_6_, it lies 0.10 eV above the CBM, whereas in MgSb_2_O_6_, it sits at 0.40 eV below the CBM. Despite a relatively higher native electron concentration in CdSb_2_O_6_, it is still insufficient to achieve metallic-like conductivity. MgSb_2_O_6_ with a low carrier concentration just above10^13^ cm^–3^ is highly insulating. Therefore, to achieve competitive conductivity, a suitable extrinsic donor dopant is required in both systems to enhance the carrier concentration while minimizing the perturbations on the CBM states and preserving good carrier mobilities.

## Ga- and F-Doped MgSb_2_O_6_

Two potential donor dopants were considered in MgSb_2_O_6_, Ga was targeted as a substituent for Mg and F was introduced on the anion sites. Ga-doping has been extensively investigated for TCO applications, including systems like Ga-doped ZnO and ZnSb_2_O_6_, where high conductivity arises from substitutional defect formation.^18,60^ Fluorine is widely used as an extrinsic dopant on oxygen sites in TCOs such as SnO_2_, ZnO, anatase TiO_2_ and BaSnO_3_ due to its electronegativity and ionic radius comparable to that of oxygen, which allows for favorable substitution with minimal lattice distortion.^56,57,61,62^ It has also been studied in the binary Sb­(V) system, Sb_2_O_5_, which shows degenerate *n*-type TCO behavior.^19^ As we cannot assume that the Ga and F simply form the target dopant defect we consider Ga_Mg_, Ga_Sb_, Ga_i_ for Ga doping and F_O_ and F_i_.

Figure 7a and b are the transition level diagrams for Ga-doped and F-doped MgSb_2_O_6_ under the metal-rich/O-poor (*n*-type) conditions. In both cases, Ga_Mg_ and F_O_ act as the lowest-energy donor defects with the (+1/0) transitions located at approximately 0.32 eV above the CBM. At the respective predicted self-consistent Fermi level positions, Ga_Mg_^+1^ has a formation energy of 1.14 eV which is around 1.1 eV lower than that of F_O_^+1^, suggesting that the formation of Ga_Mg_ would may be the more favorable in MgSb_2_O_6_. Ga_i_ has a high formation energy over 6 eV, leading to an insignificant amount of concentration and negligible contribution to the overall conductivity. Although Ga_Mg_ exhibits a low formation energy, it is charge-compensated by the acceptor defect Ga_Sb_, whose −2 charge state is stable at high Fermi levels and can readily form with a formation energy as low as 0.32 eV. This is further demonstrated in the carrier concentrations calculated as a function of annealing temperature plot in Figure S8­(b), where both Ga_Mg_ and Ga_Sb_ exhibit comparable defect concentrations of about 1 × 10^18^ cm^–3^. However, the compensating effect of Ga_Sb_ offsets the electron contributions from the other donor defects, limiting the overall electron concentration to ∼ 1 × 10^17^ cm^–3^.

**Figure 7:**
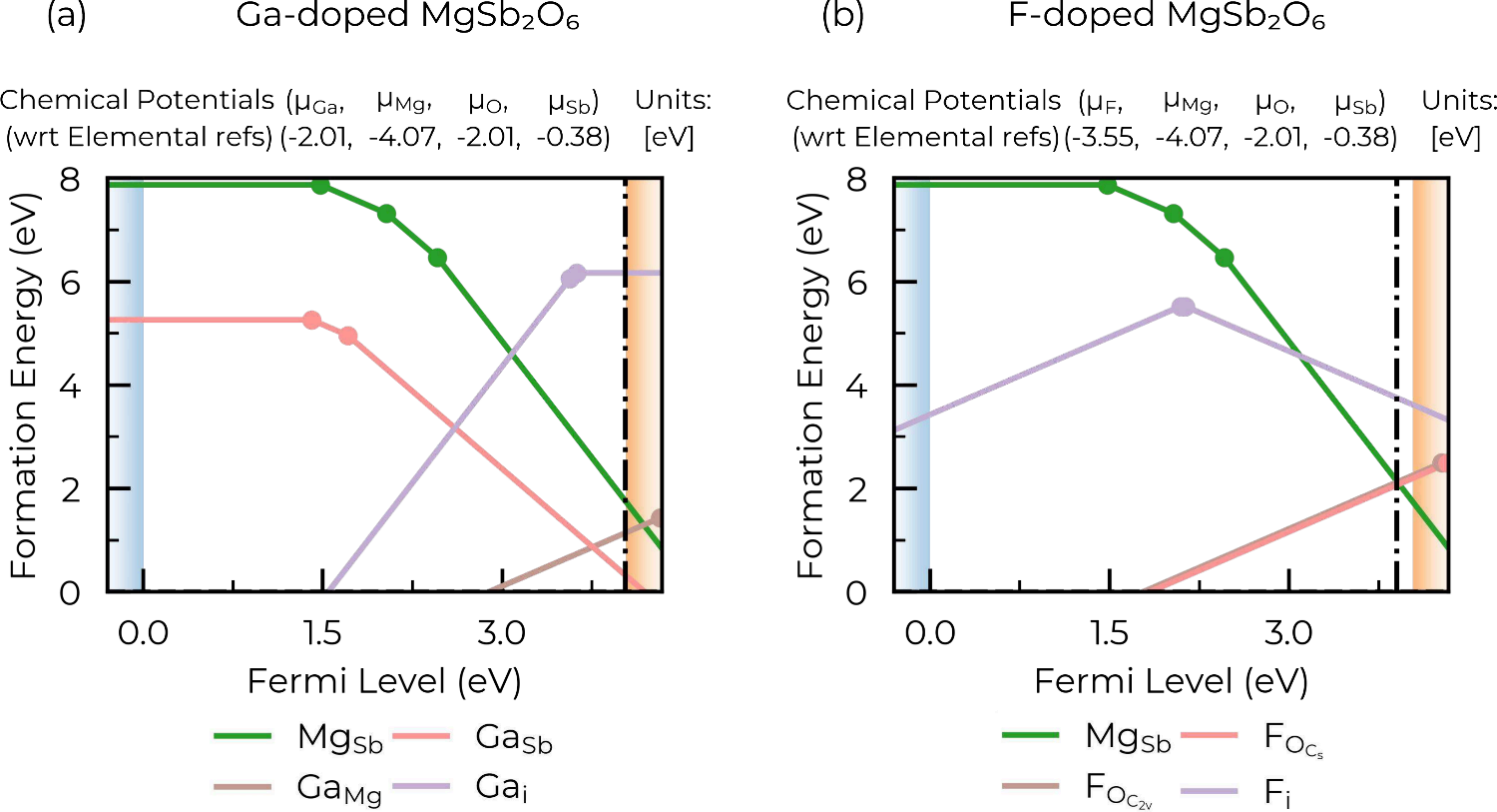
(a) Transition level diagram of the extrinsic defects with the intrinsic point defect that sets the *n*-type doping window in Ga-doped MgSb_2_O_6_ under metal-rich/O-poor conditions.^39^ The calculated self-consistent Fermi level (black dotted line) lies at the conduction band edge at an annealing temperature of 1000 K.^39,44^ (b) Transition level diagram of the extrinsic defects with the intrinsic point defect that sets the *n*-type doping window in F-doped MgSb_2_O_6_ under metal-rich/O-poor conditions.^39^ The calculated self-consistent Fermi level (black dotted line) is located 0.1 eV under the CBM at an annealing temperature of 1000 K.^39,44^

In F-doped MgSb_2_O_6_, F_i_^+1^ is stable across half the Fermi level range as an energy-lowering structure with an 1.43 Å O–O dimer is formed. Mg_Sb_ continues to charge-compensate the donor effect of F_O_, residing the self-consistent Fermi level within the band gap. Therefore, the impact of F_O_ on the overall conductivity remains limited due to its relatively high formation energy and the charge-compensating effect from Mg_Sb_. The resulting electron concentration is increased to 1 × 10^15^ cm^–3^ as shown in Figure S9­(b). While both Ga and F-doping in MgSb_2_O_6_ are found to be insufficient in achieving degenerate *n*-type conductivity, the self-consistent Fermi levels are positioned on and close to the CBM, and the overall carrier concentrations are enhanced by 2–3 orders of magnitude compared to those generated from the intrinsic point defects. Considering temperature-dependent band gap narrowing and the effects of higher annealing temperatures, even higher carrier concentrations may be attainable.

## Y-Doped CdSb_2_O_6_

To increase the carrier concentrations in CdSb_2_O_6_, Y was selected as an extrinsic dopant, given that Y^3+^ has previously been employed to substitute Cd^2+^ in other cadmium-based oxides such as CdO and Cd_2_SnO_4_ to enhance *n*-type conductivity.^63–65^ The similar ionic radii of Y^3+^ (0.90 Å) and Cd^2+^ (0.95 Å) could allow for the formation of low energy Y_Cd_^+1^ species, acting as donors and indeed previous experimental literature shows Y-doped CdSb_2_O_6_ exhibits carrier concentrations of 1 × 10^20^ cm^–3^, yielding a moderate conductivity.^16,66^ Figure 8(a) is the transition level diagram of the extrinsic defects in Y-doped CdSb_2_O_6_ under metal-rich/O-poor conditions, where the limiting phase is YSbO_4_. Among all the extrinsic defects, Y_Cd_ is the lowest-energy donor, which acts as a resonant defect with (+1/0) transition at around 0.56 eV above the CBM. The resulting YO_6_ octahedra have a shorter Y–O bond length of 2.32 Å compared to the 2.37 Å Cd–O bond length observed in the bulk CdO_6_ octahedra. Y_i_ has a very high formation energy of around 7.90 eV at the predicted self-consistent Fermi level position indicated as the black dotted line.

**Figure 8:**
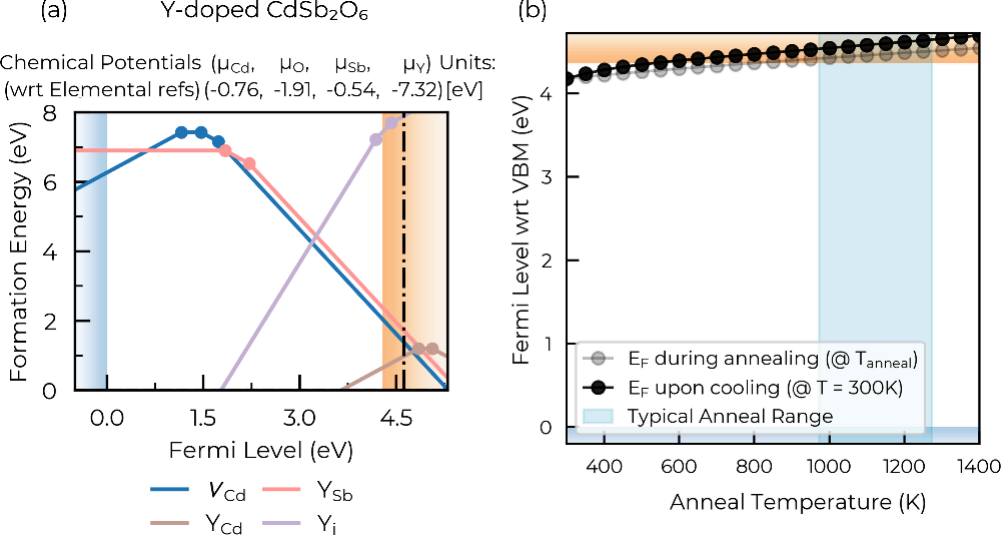
(a) Transition level diagram of the extrinsic defects with the intrinsic point defect that sets the *n*-type doping window in Y-doped CdSb_2_O_6_ under metal-rich/O-poor conditions.^39^ The predicted self-consistent Fermi level is located 0.33 eV above the CBM at an annealing temperature of 1200 K.^39,44^ (b) Calculated Fermi level positions as a function of annealing temperature. Gray points represent the Fermi level during annealing, while black points indicate the predicted Fermi level position after cooling to room temperature (300 K). The shaded blue region corresponds to the typical annealing temperature range used in experiments.^39^

Figure 8(b) illustrates the changes in the predicted self-consistent Fermi level positions as a function of annealing temperature under Y-doping. The calculated self-consistent Fermi levels upon cooling are located above the CBM within the typical annealing temperature range, indicating its degenerate conductivity. The related defect and electron concentrations are shown in Figure 9(a). Y_Cd_, being the lowest-energy donor defect, is the dominant contributor to the *n*-type conductivity, with its concentration matching the overall electron concentration of above 1 × 19^20^ cm^–3^. The resonant defect nature of Y_Cd_ in its fully ionized charge state is evident in the unfolded band structure shown in Figure 9. The Y 4*d* states appear at approximately 2.5 eV above the CBM, with localized “bands” lying ∼ 5.0 eV above the CBM. No hybridization with the CBM upon Y-doping indicates negligible perturbation to the CBM, suggesting that high mobilities can be preserved. This behavior is analogous to other resonant dopants in conventional TCOs such as Ta_Sn_ and W_Sn_ in SnO_2_, Mo_In_ in In_2_O_3_, and La_Ba_ in BaSnO_3_.^21,22,56,67,68^ Overall, yttrium acts as an effective dopant by forming Y_Cd_ as a low-energy, resonant donor defect, resulting in a high electron concentration exceeding 1 × 19^20^ cm^–3^ and showing degenerate transparent conducting behavior.

**Figure 9:**
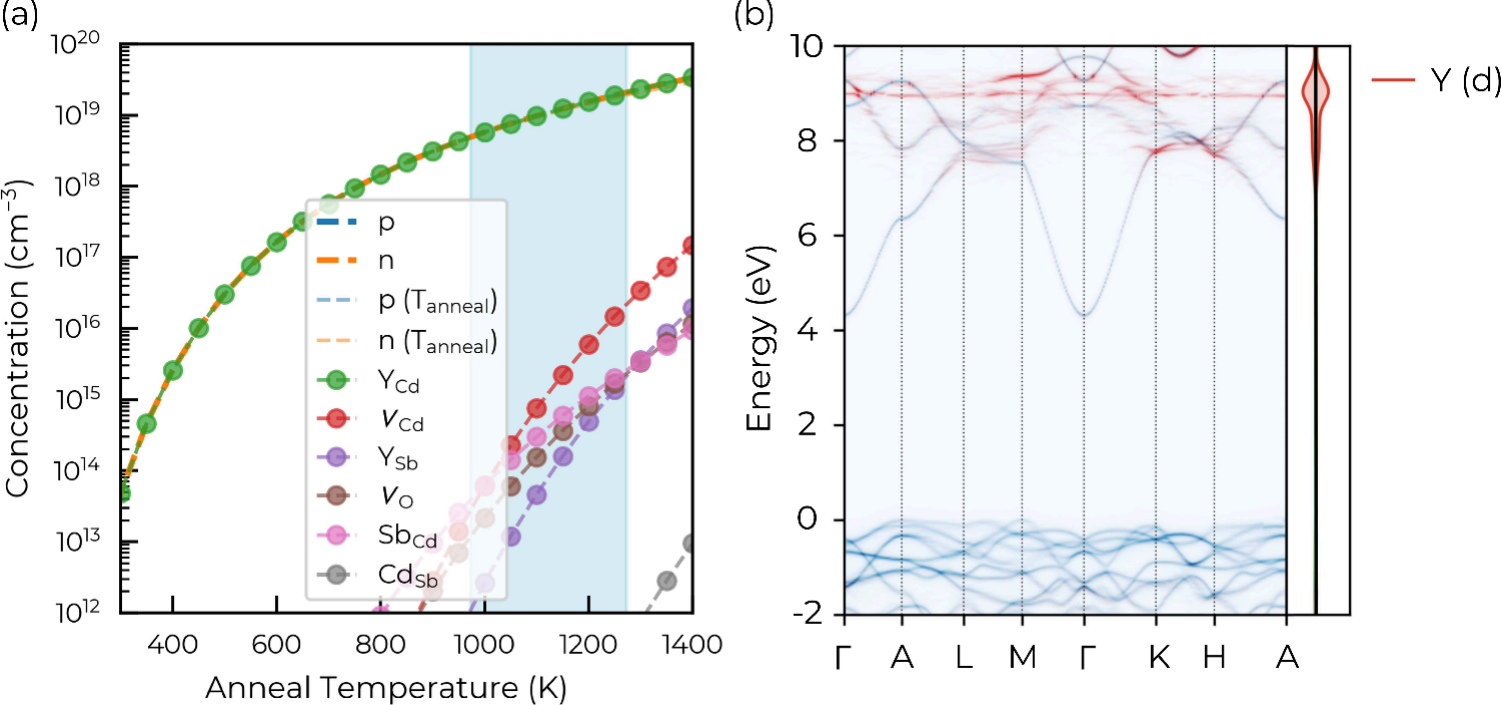
(a) Defect carrier concentrations of Y-doped CdSb_2_O_6_ as a function of annealing temperature. Defects with concentrations lower than 1 × 12^20^ cm^–3^ have been omitted.^39^ (b) Projected unfolded band structure of Y_Cd_ in the fully ionized charge state (+1) with the density of states of the Y 4d orbital shown.

This result indicates that the moderate conductivity found in previous studies of Y-doped CdSb_2_O_6_ likely stems from poor crystallinity or grain boundary effects,^16,66^ as our results indicate that high conductivity and high mobility are possible for Y-doped CdSb_2_O_6_. It should be noted that donor-doped BaSnO_3_ was ruled out as a promising TCO for decades until single crystals demonstrated that it possessed the highest mobility of any *n*-type TCO. Therefore, caution should always be taken when ruling a material out of contention, unless high quality single crystal studies demonstrate poor performance.

## Band Alignments

Figure 10 shows the calculated ionization potential (IP) and electron affinity (EA) for the Sb­(V)-based oxides, and the comparison with different state-of-the-art TCOs.^55,69,70^ The ASb_2_O_6_ family exhibits notably high IPs with values close to or exceeding 9.6 eV, significantly deeper than the other conventional TCOs. However, the EA values vary across the series where MgSb_2_O_6_ and CdSb_2_O_6_ show relatively large electron affinities (5.8 and 5.4 eV, respectively), while the remaining species exhibit lower EAs in the range of 3.8 to 4.1 eV. The discrepancy between the EAs of MgSb_2_O_6_ and CdSb_2_O_6_ and the other Sb­(V)-oxides can be attributed to structural and electrostatic effects introduced by the A-site cation. The CBM is mainly comprised of Sb 5*s* orbitals, where due to the high oxidation state of Sb^5+^, are held closer to the nucleus, experiencing reduced shielding and stronger nuclear attraction compared to +3/+2 post-transition cations. However, in compositions with larger A-site cations (Ca^2+^, Sr^2+^, Ba^2+^), the expanded lattice and reduced electrostatic potential near the Sb centers lead to a shallower CBM, i.e., one positioned closer to the vacuum level, thereby reducing the electron affinity. In contrast, smaller A-site cations such as Mg^2+^ and Cd^2+^ enhance the local electrostatic attraction and Sb 5*s*-O 2*p* orbital overlap, pushing the CBM to deeper energies relative to the vacuum level and resulting in higher electron affinities.

**Figure 10:**
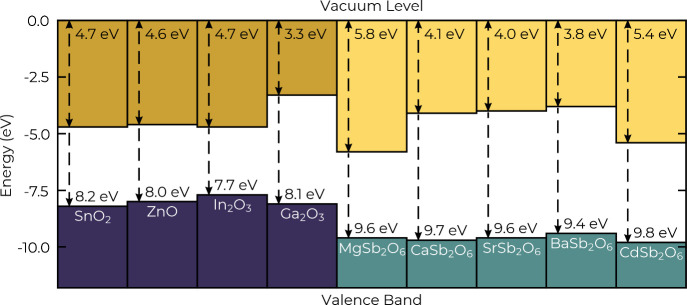
Calculated band alignments for the different ASb_2_O_6_ compared to those of two state-of-art TCOs.^55,69,70^

## Experimental Validation of CdSb_2_O_6_ and Y-Doped CdSb_2_O_6_

The PXRD pattern of CdSb_2_O_6_ fitted with a model in the *P* 31*m* space group is shown in Figure S11­(a). The lattice parameters obtained by Rietveld Refinement were a = 5.237(5) and c = 4.7993(7) Å, Volume = 113.9 Å^3^ very close to the reported values of Castro et al.^47^ and our computational predictions. Doping with 9.1% Y led to a modest change in lattice volume (Figure S11­(b)), with parameters a = 5.235(5) and c = 4.804(3) Å, Volume = 114.1 Å^3^, and the detection of a secondary Y_2_O_3_ phase. Given the very similar ionic radii of Y^3+^ and Cd_2_^+^, the small change in lattice parameters is not surprising. The optical band gap was obtained through a Tauc plot from diffuse reflectance spectra is shown in Figure 11(a). A shift of the band gap with doping can be seen, increasing from 3.48 eV in the undoped material to 3.71 eV in the doped material. This increase is consistent with the Burstein–Moss (BM) shift expected on *n*-doping of a semiconductor.

**Figure 11:**
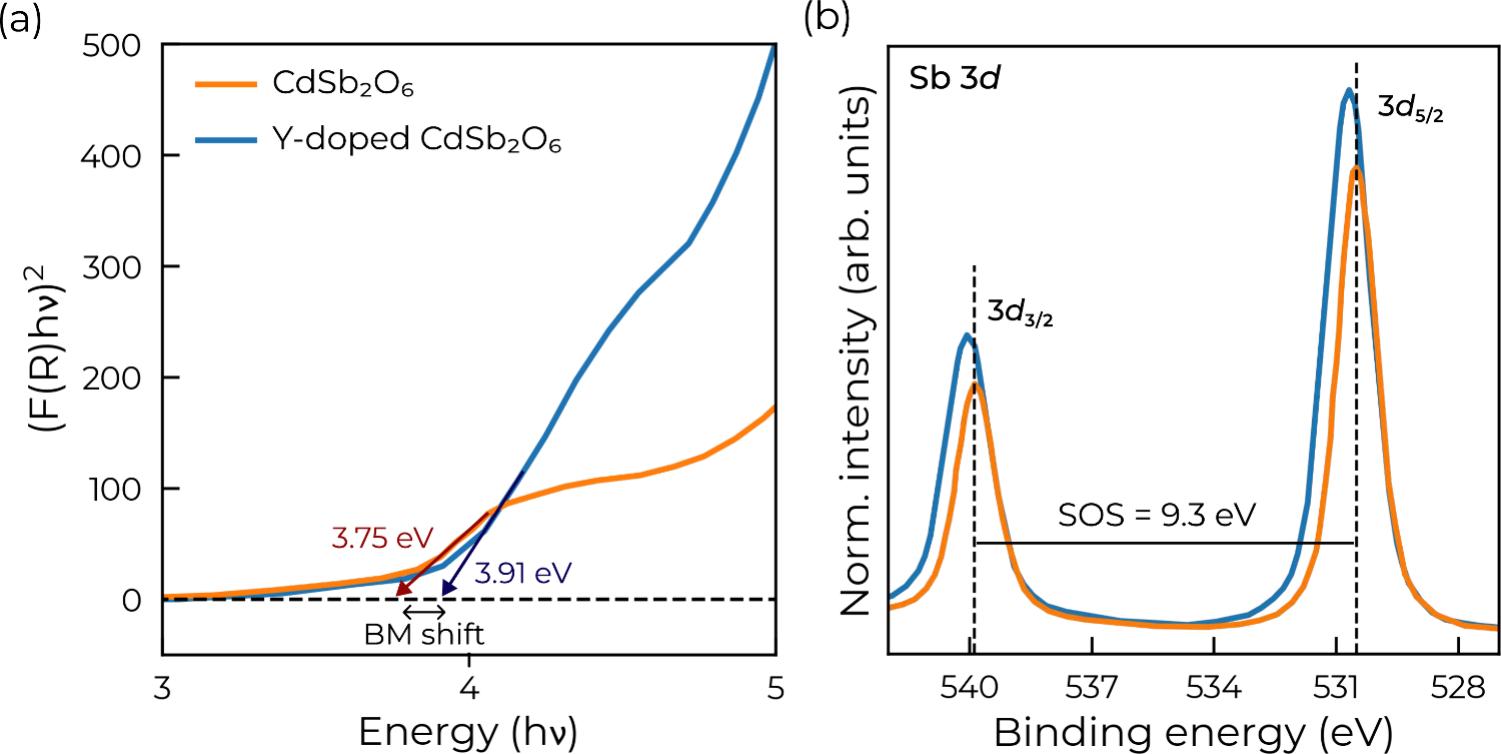
(a) Experimental Tauc plot for CdSb_2_O_6_ and Y-doped CdSb_2_O_6_ obtained with UV–vis measurements. The Burstein–Moss (MB) shift is shown. (b) XP spectra comparing the position of the Sb 3d_3/2_ and 3d_5/2_ peak of undoped and Y-doped CdSb_2_O_6_.

As presented in Figure 11(b), XP spectra taken from the undoped compound showed Sb 3*d*_3/2_ peak (chosen due to the overlap of the Sb 3*d*_5/2_) peak with O 1*s*) at 540.2 eV, and the Cd 3*d*_5/2_ at 404.9 eV. The Y-doped sample has a shift of around 0.1 eV or all spectral features to higher binding energy, again consistent with an increase in the Fermi level that occurs during *n*-type doping.

## Conclusion

In summary, we have conducted a screening study of different ASb_2_O_6_ compounds, evaluating their optical properties and carrier mobility. Our results predict that all of these oxides are optically transparent and possess interesting mobilities for TCO applications. Within this series, MgSb_2_O_6_ and CdSb_2_O_6_ emerge as the most promising candidates, displaying high mobility (in the range of state-of-the-art TCOs) in both low and high carrier concentration regimes. Intrinsic defect study reveals that although undoped MgSb_2_O_6_ and CdSb_2_O_6_ are insulating and weakly *n*-type, their wide doping windows are favorable for donor incorporation. Ga and F-doping in MgSb_2_O_6_ enhanced the carrier concentrations to 2–3 orders of magnitude compared to the intrinsic level. Y-doped CdSb_2_O_6_ forms a low-energy resonant defect (Y_Cd_) with donor *d*-states lying well above the conduction band minimum, enabling high carrier concentration while preserving high mobility. Experimental Tauc plot derived from diffuse reflectance spectra shows an increase in the optical band gap upon Y incorporation, consistent with the Burstein–Moss shift expected for *n*-type doping. XPS measurements display a ∼ 0.1 eV shift of core-level features to higher binding energies in the Y-doped sample, indicating a Fermi-level increase upon Y-doping, in agreement with the computational predictions. Finally, the unique band alignments of these Sb­(V) oxides, relative to the classic state-of-the-art TCOs, open the door to unexplored, high-performance device-integration strategies.

## Supplementary Material



## Notes

Additional computational and experimental data, including phonon-limited mobility convergence testing, AMSET transport parameters and mobility analysis, structural and electronic properties, competing phase formation energies and chemical potential limits, self-consistent Fermi level calculations, phonon band structures, and experimental characterization of pristine and Y-doped CdSb_2_O_6_ using PXRD, XPS, and optical measurements (PDF)
