# Supplementary material for: Screening ASb2O6 (A = Mg, Ca, Sr, Ba, or Cd) for High-Performance Transparent Conducting Oxides
Source: Chem Mater. 2026 Jun 12;38(13):6698–710. doi: 10.1021/acs.chemmater.6c00854 (PMC13374008; doi:10.1021/acs.chemmater.6c00854)
Supplement: Supplementary file 1 [file cm6c00854_si_001.pdf]

Screening ASb_2O_6 (A = Mg, Ca, Sr, Ba, Cd) for High Performance Transparent Conducting Oxides

Romain Claes,[†,‡] Ke Li,[¶,‡] Fatima, Sajid,[¶] Alexander G. Squires,[†] Robert G. Palgrave,[¶] and David O. Scanlon[*,†]

†*School of Chemistry, University of Birmingham, Edgbaston, Birmingham, B15 2TT, UK*

‡*These authors contributed equally to this work*

¶*Department of Chemistry, University College London, 20 Gordon Street, London WC1H 0AJ, UK*

E-mail: d.o.scanlon@bham.ac.uk

Phone: +44 (0)20 7679 4558

Supplementary Information contains phonon dispersion, charge transport constants, further AMSET plots and some convergence testing and defect formation energy data.

Electron Transport

Phonon-limited mobility convergence (computed with ABINIT)

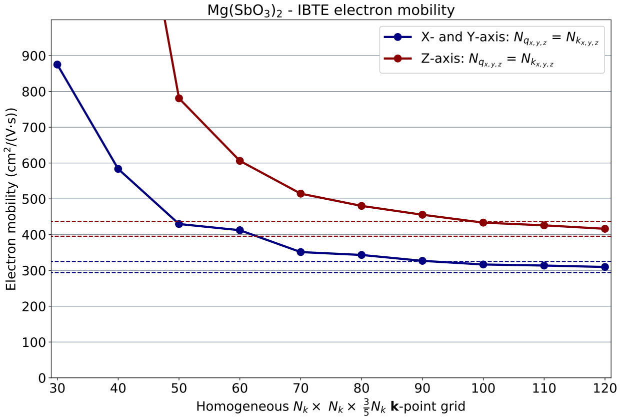

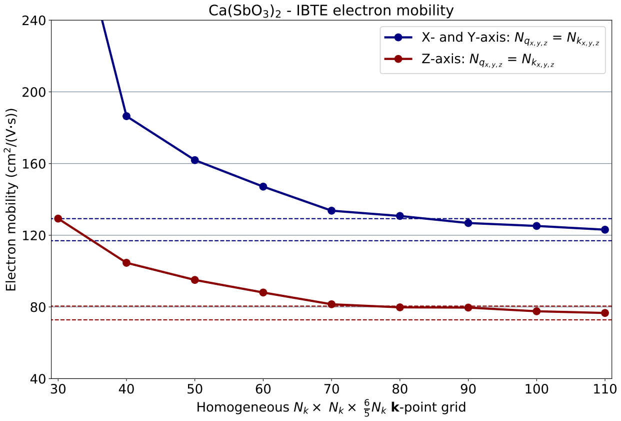

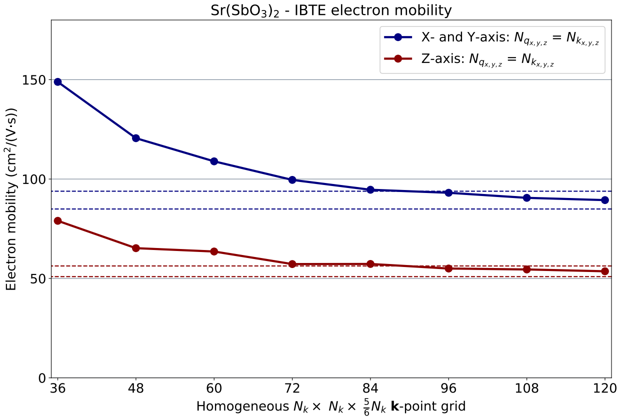

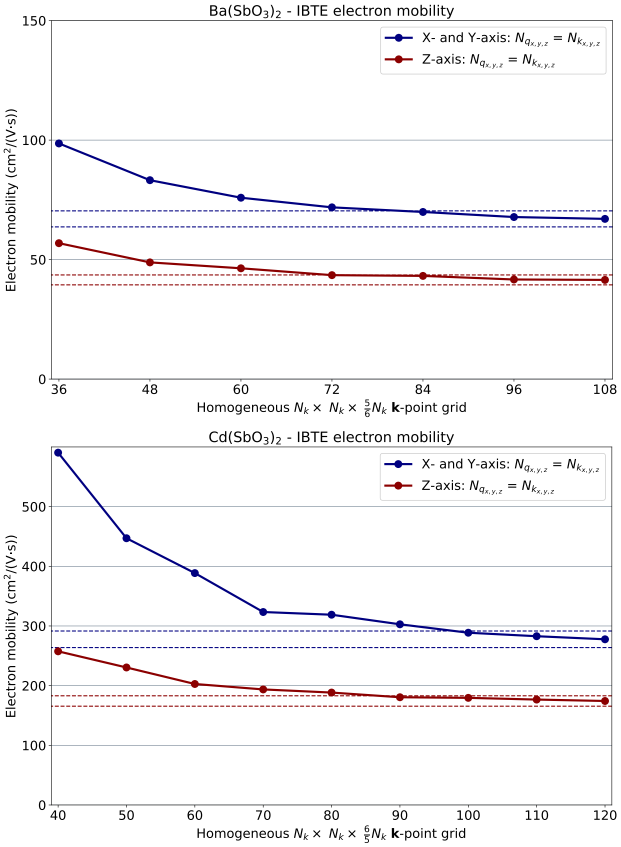

Figure 1: IBTE hole mobility as a function of the **k**- and **q**-point grids at 300K for the different Sb(V) oxides. A convergence is assumed when 3 consecutive points are within a 5% difference.

Table 1: Comparison of the electron mobility with and without the inclusion of dynamical quadrupoles (Q^*). In the case of Sb(V) oxides, the difference is clearly negligible.

Materials	Without Q* ($cm^2\,V^{-1}\,s^{-1}$)		With Q* ($cm^2\,V^{-1}\,s^{-1}$)		Difference (%)	
	a- and *b*-axis	*c*-axis	*a*- and *b*-axis	*c*-axis	*a*- and *b*-axis	*c*-axis
$MgSb_2O_6$	309.66	416.30	309.04	419.90	0.2	0.87
$CdSb_2O_6$	277.67	174.27	278.20	174.23	0.19	0.02

AMSET inputs

$\mathbf{MgSb_2O_6}$

Polar optical phonon frequency: 13.13 THz

Elastic constant (DFPT):

$$\begin{bmatrix} 520.5 & 239.3 & 195.1 & 0 & 0 & 0 \\ 239.3 & 520.8 & 195.2 & 0 & 0 & 0 \\ 195.1 & 195.2 & 658.8 & 0 & 0 & 0 \\ 0 & 0 & 0 & 196.3 & 0 & 0 \\ 0 & 0 & 0 & 0 & 194.3 & 0 \\ 0 & 0 & 0 & 0 & 0 & 298.5 \end{bmatrix}$$

High-frequency dielectric constant (ε_∞):

$$\begin{bmatrix} 2.95 & 0 & 0 \\ 0 & 2.95 & 0 \\ 0 & 0 & 3.22 \end{bmatrix}$$

Low-frequency dielectric constant (ε_{low}):

$$\begin{bmatrix} 7.25 & 0 & 0 \\ 0 & 7.25 & 0 \\ 0 & 0 & 4.6 \end{bmatrix}$$

Total (static) dielectric constant ($\varepsilon_{\text{static}}$):

$$\begin{bmatrix} 10.20 & 0 & 0 \\ 0 & 10.20 & 0 \\ 0 & 0 & 7.82 \end{bmatrix}$$

$CaSb_2O_6$

Polar optical phonon frequency: 10.72 THz

Elastic constant (DFPT):

$$\begin{bmatrix} 471.8 & 231.2 & 186.0 & 0 & 0 & 0 \\ 231.2 & 471.9 & 186.1 & 0 & 0 & 0 \\ 186.0 & 186.1 & 616.7 & 0 & 0 & 0 \\ 0 & 0 & 0 & 174.6 & 0 & 0 \\ 0 & 0 & 0 & 0 & 173.5 & 0 \\ 0 & 0 & 0 & 0 & 0 & 265.1 \end{bmatrix}$$

High-frequency dielectric constant (ε_∞):

$$\begin{bmatrix} 3.03 & 0 & 0 \\ 0 & 3.03 & 0 \\ 0 & 0 & 3.23 \end{bmatrix}$$

Low-frequency dielectric constant (ε_{low}):

$$\begin{bmatrix} 6.38 & 0 & 0 \\ 0 & 6.38 & 0 \\ 0 & 0 & 3.9 \end{bmatrix}$$

Total (static) dielectric constant ($\varepsilon_{\text{static}}$):

$$\begin{bmatrix} 9.41 & 0 & 0 \\ 0 & 9.41 & 0 \\ 0 & 0 & 7.13 \end{bmatrix}$$

$SrSb_2O_6$

Polar optical phonon frequency: 10.64 THz

Elastic constant (DFPT):

$$\begin{bmatrix} 421.6 & 214.4 & 165.6 & 0 & 0 & 0 \\ 214.4 & 421.7 & 165.6 & 0 & 0 & 0 \\ 165.6 & 165.6 & 569.8 & 0 & 0 & 0 \\ 0 & 0 & 0 & 154.2 & 0 & 0 \\ 0 & 0 & 0 & 0 & 151.7 & 0 \\ 0 & 0 & 0 & 0 & 0 & 235.4 \end{bmatrix}$$

High-frequency dielectric constant (ε_∞):

$$\begin{bmatrix} 3.13 & 0 & 0 \\ 0 & 3.13 & 0 \\ 0 & 0 & 3.29 \end{bmatrix}$$

Low-frequency dielectric constant (ε_{low}):

$$\begin{bmatrix} 5.84 & 0 & 0 \\ 0 & 5.84 & 0 \\ 0 & 0 & 3.51 \end{bmatrix}$$

Total (static) dielectric constant ($\varepsilon_{\text{static}}$):

$$\begin{bmatrix} 8.97 & 0 & 0 \\ 0 & 8.97 & 0 \\ 0 & 0 & 6.80 \end{bmatrix}$$

$BaSb_2O_6$

Polar optical phonon frequency: 10.21 THz

Elastic constant (DFPT):

$$\begin{bmatrix} 384.0 & 197.3 & 151.3 & 0 & 0 & 0 \\ 197.3 & 384.1 & 151.4 & 0 & 0 & 0 \\ 151.3 & 151.4 & 531.6 & 0 & 0 & 0 \\ 0 & 0 & 0 & 138.3 & 0 & 0 \\ 0 & 0 & 0 & 0 & 137.3 & 0 \\ 0 & 0 & 0 & 0 & 0 & 214.8 \end{bmatrix}$$

High-frequency dielectric constant (ε_∞):

$$\begin{bmatrix} 3.21 & 0 & 0 \\ 0 & 3.21 & 0 \\ 0 & 0 & 3.33 \end{bmatrix}$$

Low-frequency dielectric constant ($\varepsilon_{\mathrm{low}}$):

$$\begin{bmatrix} 5.59 & 0 & 0 \\ 0 & 5.59 & 0 \\ 0 & 0 & 3.35 \end{bmatrix}$$

Total (static) dielectric constant ($\varepsilon_{\mathrm{static}}$):

$$\begin{bmatrix} 8.80 & 0 & 0 \\ 0 & 8.80 & 0 \\ 0 & 0 & 6.68 \end{bmatrix}$$

$CdSb_2O_6$

Polar optical phonon frequency: 13.30 THz

Elastic constant (DFPT):

$$\begin{bmatrix} 524.4 & 241.0 & 194.8 & 0 & 0 & 0 \\ 241.0 & 524.4 & 194.8 & 0 & 0 & 0 \\ 194.8 & 194.8 & 672.0 & 0 & 0 & 0 \\ 0 & 0 & 0 & 194.7 & 0 & 0 \\ 0 & 0 & 0 & 0 & 192.9 & 0 \\ 0 & 0 & 0 & 0 & 0 & 297.2 \end{bmatrix}$$

High-frequency dielectric constant (ε_∞):

$$\begin{bmatrix} 3.04 & 0 & 0 \\ 0 & 3.04 & 0 \\ 0 & 0 & 3.25 \end{bmatrix}$$

Low-frequency dielectric constant (ε_{low}):

$$\begin{bmatrix} 6.39 & 0 & 0 \\ 0 & 6.39 & 0 \\ 0 & 0 & 4.14 \end{bmatrix}$$

Total (static) dielectric constant ($\varepsilon_{\text{static}}$):

$$\begin{bmatrix} 9.43 & 0 & 0 \\ 0 & 9.43 & 0 \\ 0 & 0 & 7.39 \end{bmatrix}$$

Electron mobility results with different approaches

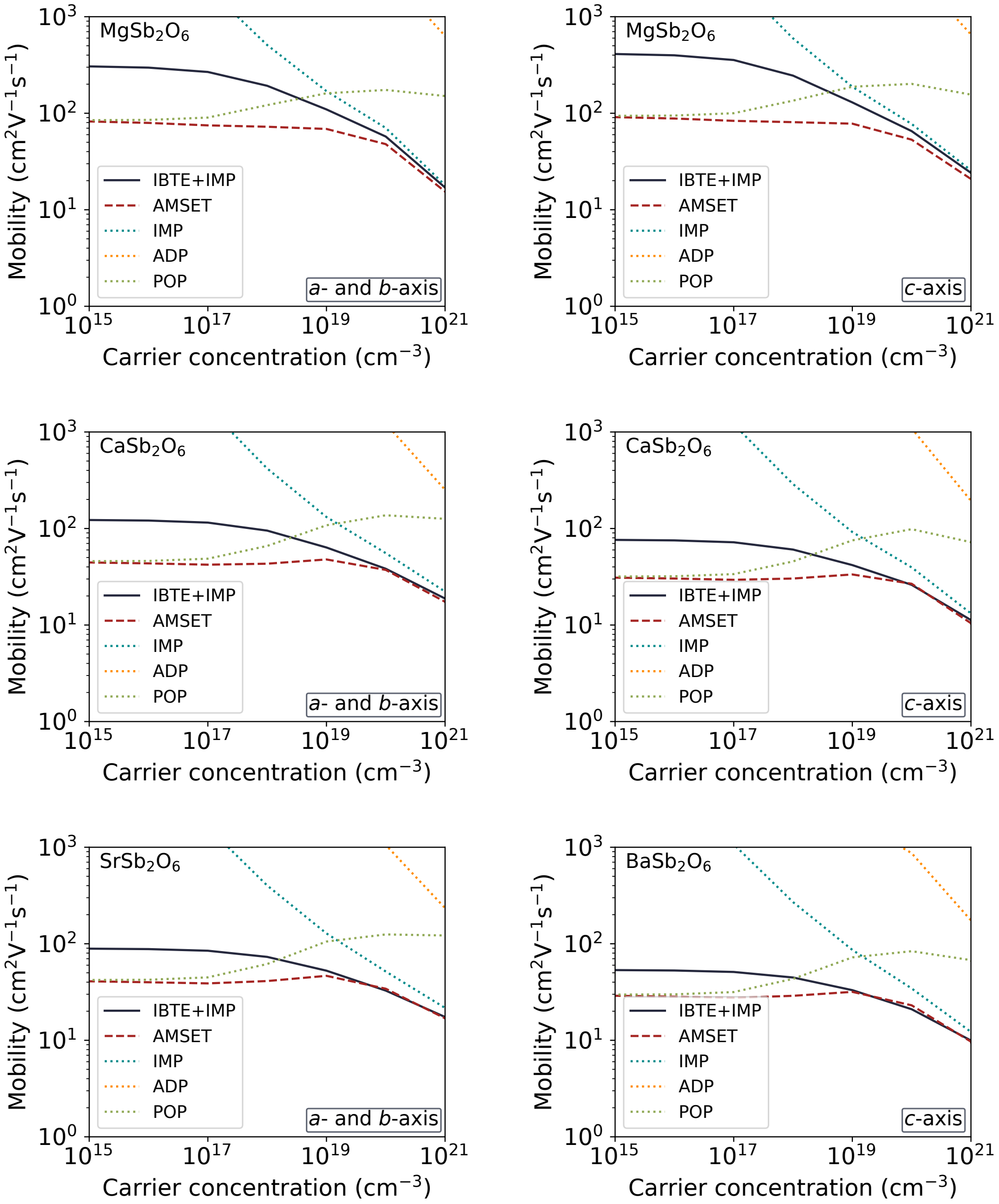

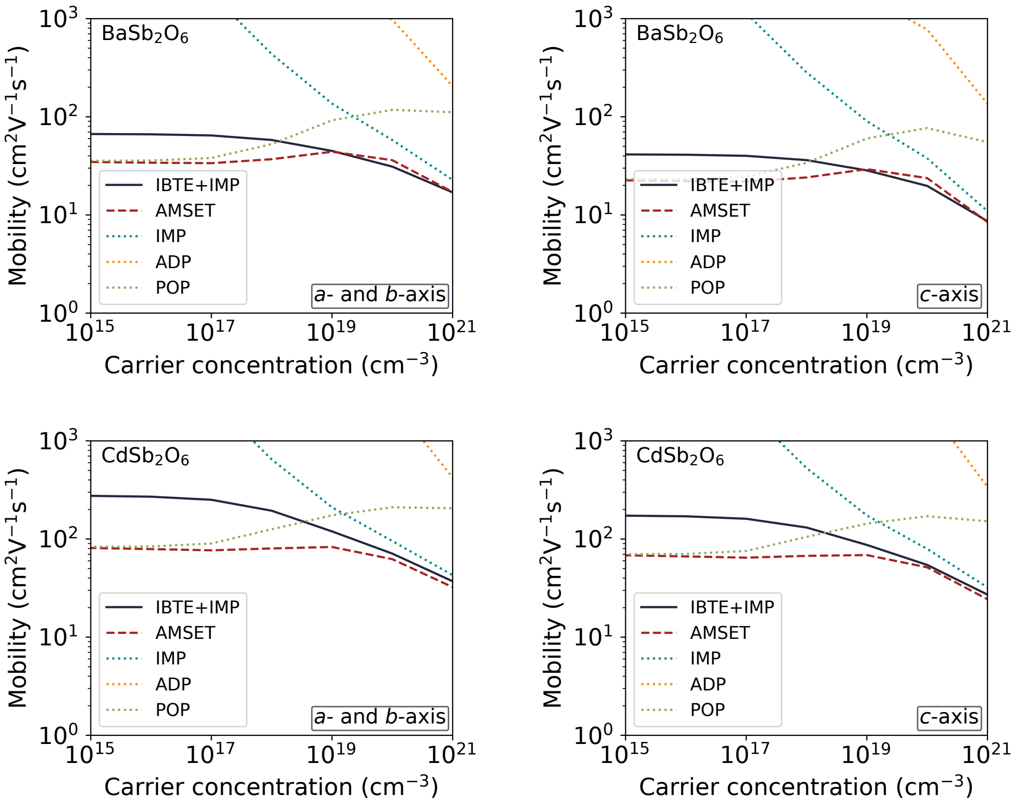

Figure 2: Computed mobility against the carrier concentration of the different Sb(V) oxides along the different axis. In these figures, IBTE stands for the iterative Boltzmann transport equation phonon-limited mobility computed with ABINIT, AMSET for the total mobility computed with AMSET, IMP for impurity scattering, POP for polar-optical scattering, and ADP for acoustic deformation potential scattering.

Additional crystal properties

Table 2: Structural properties of the different ASb_2O_6 compounds studied in this work.

Materials	A–O distances (Å)	
	PBE0	Exp.
$MgSb_2O_6$	2.047 & 2.063	2.012 & 2.050 [1]
$CaSb_2O_6$	2.415	2.437 [2]
$SrSb_2O_6$	2.558	2.58 [2]
$BaSb_2O_6$	2.728	2.745 [2]
$CdSb_2O_6$	2.355	2.344 [1]

Electronic band structures

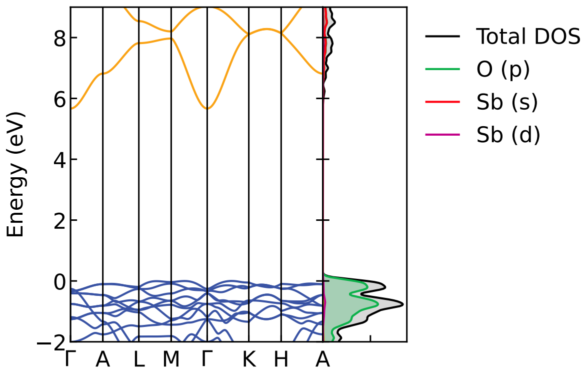

(a) $CaSb_2O_6$

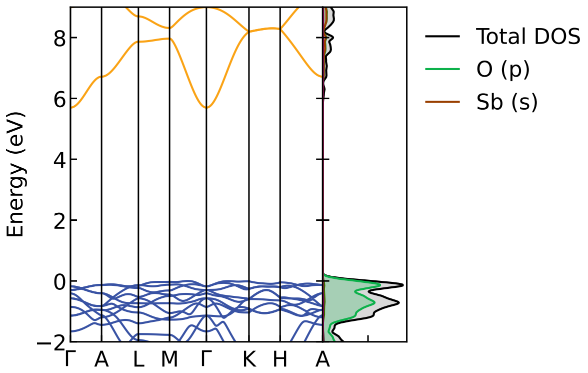

(b) $SrSb_2O_6$

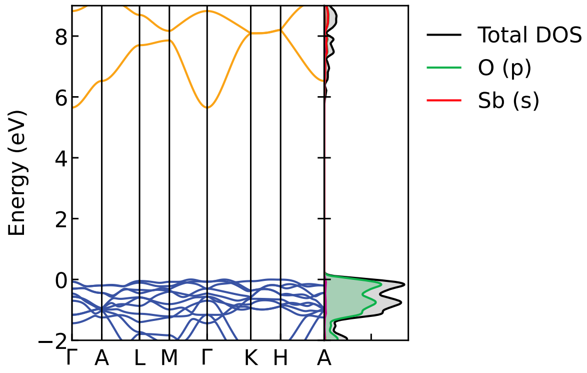

(c) $BaSb_2O_6$

Figure 3: Electronic band structure of Ca, Sr and $BaSb_2O_6$ computed with the PBE0 functional.

Intrinsic Defect Chemistry

Table 3: Formation energies per formula unit (ΔE_f) of $MgSb_2O_6$ and all competing phases, with k-meshes used in calculations. Only the lowest energy polymorphs are included.

Formula	Space Group	k-mesh	ΔE_f (eV/fu)
$MgSb_2O_6$	$P4_2/mnm$	6×6×4	-16.886
Mg	$R\bar{3}m$	17×17×17	0.000
Sb	$R\bar{3}m$	11×11×11	0.000
O_2	P4/mmm	1×1×1	0.000
Sb_2O_3	$Fd\bar{3}m$	4×4×4	-6.791
Sb_2O_5	C2/c	6×6×5	-9.321
SbO_2	Pnna	4×3×1	-4.294
$MgSb_2O_4$	$P4_2/mbc$	5×5×7	-12.867
$Mg_4Sb_2O_9$	$P\bar{1}$	6×6×3	-34.118

Table 4: Chemical potential limits for $MgSb_2O_6$.

**Limit**	**Mg**	**Sb**	**O**
$Mg(SbO_3)_2$-$Mg_4Sb_2O_9$-O_2	-5.74	-5.57	0.00
Sb_2O_5-$Mg(SbO_3)_2$-O_2	-7.57	-4.66	0.00
Sb_2O_5-$Mg(SbO_3)_2$-SbO_2	-6.83	-2.83	-0.73
$Mg(SbO_2)_2$-$Mg(SbO_3)_2$-$Mg_4Sb_2O_9$	-3.73	-0.55	-2.01
Sb_2O_3-$Mg(SbO_3)_2$-SbO_2	-4.70	-0.70	-1.80
Sb_2O_3-$Mg(SbO_2)_2$-$Mg(SbO_3)_2$	-4.07	-0.38	-2.01

Table 5: Formation energies per formula unit (ΔE_f) of $CdSb_2O_6$ and all competing phases, with k-meshes used in calculations. Only the lowest energy polymorphs are included.

Formula	Space Group	k-mesh	ΔE_f (eV/fu)
$CdSb_2o_6$	P$\overline{3}$1m	5×5×6	-13.292
Cd	P6_3/mmc	14×14×7	0.000
Sb	R$\overline{3}$m	11×11×11	0.000
O_2	Pmmm	1×1×1	0.000
CdO	Fm$\overline{3}$m	7×7×7	-2.156
Sb_2O_3	Fd$\overline{3}$m	4×4×4	-6.803
Sb_2O_5	C2/c	6×6×5	-9.344
SbO_2	Pnna	4×3×1	-4.302
$Cd_2Sb_2O_7$	Fd$\overline{3}$m	4×4×4	-15.959
CdO_2	Pa$\overline{3}$	2×2×2	-1.778

Table 6: Chemical potential limits for $CdSb_2O_6$.

**Limit**	**Cd**	**Sb**	**O**
$Cd(SbO_3)_2$-$Cd_2Sb_2O_7$-O_2	-2.67	-5.31	0.00
$Cd(SbO_3)_2$-Sb_2O_3-SbO_2	-1.08	-0.70	-1.80
$Cd(SbO_3)_2$-Sb_2O_3-$Cd_2Sb_2O_7$	-0.76	-0.54	-1.91
Sb_2O_5-$Cd(SbO_3)_2$-O_2	-3.95	-4.67	0.00
Sb_2O_5-$Cd(SbO_3)_2$-SbO_2	-3.21	-2.82	-0.74

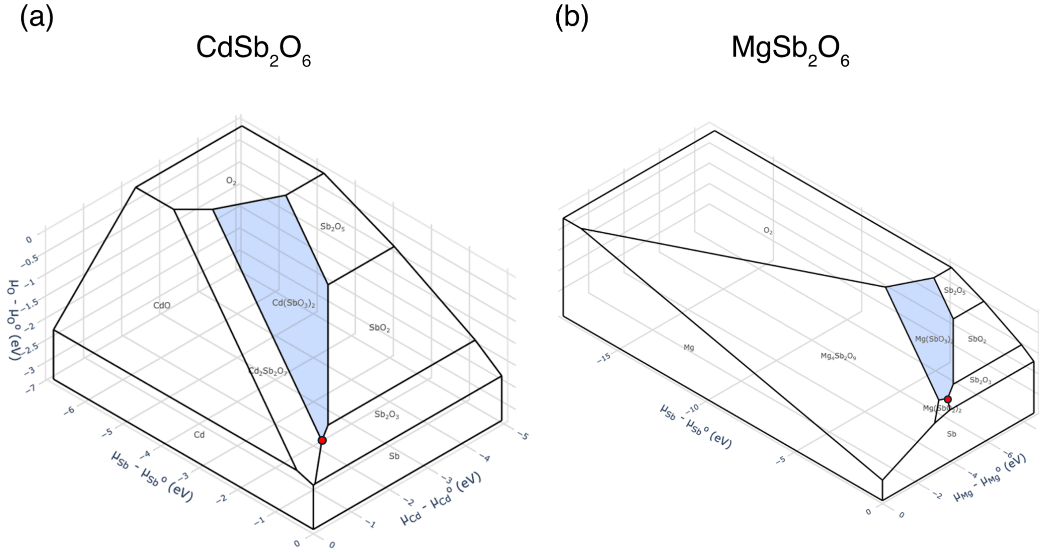

Figure 4: Phase diagrams of (a) $CdSb_2O_6$ and (b) $MgSb_2O_6$ shaded in blue. The red dot marks the most n-type (metal-rich/O-poor) growth conditions.

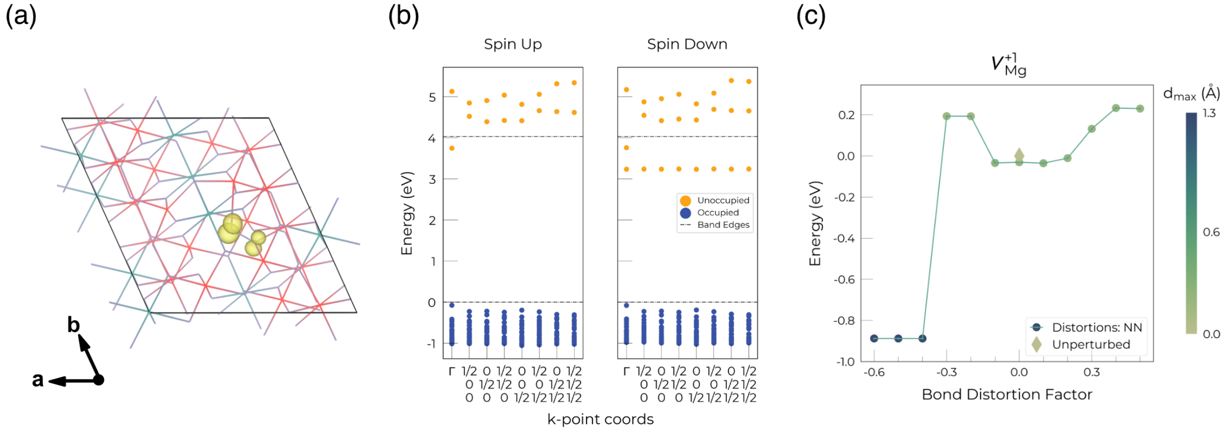

Figure 5: (a) Charge density isosurface of the hole localization on the O-O dimer formed in ${V_{\mathrm{Mg}}}^{+1}$, displayed in a wireframe structure, with isosurface level of 0.032 e Å^{-3}. (b) Eigenvalue analysis of ${V_{\mathrm{Mg}}}^{+1}$ in spin-up and spin-down channels. Unoccupied and occupied states are shown in orange and blue dots, respectively. (c) ShakeNBreak plot of energy versus bond distortion factors where the ground-state structure is found in -0.4 (-40%) distortion.

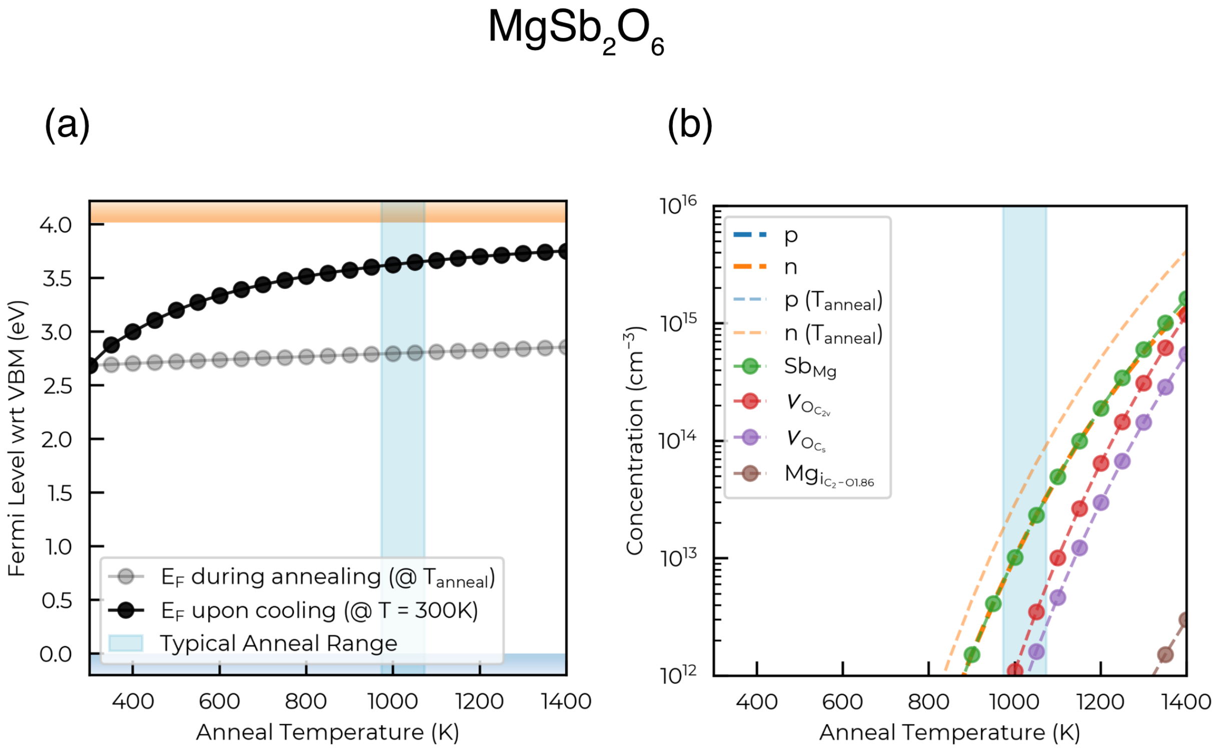

Figure 6: (a) Temperature-dependent self-consistent Fermi level positions in undoped $MgSb_2O_6$, with the grey line represents the E_F during annealing while the black line indicates the E_F after cooling to room temperature (300 K). The light blue shaded region shows the reported annealing temperature range.[3–5] (b) Plot of room-temperature intrinsic carrier and defect concentrations as a function of annealing temperatures, where the defects with concentration over 10^{12} cm^{-3} are shown.

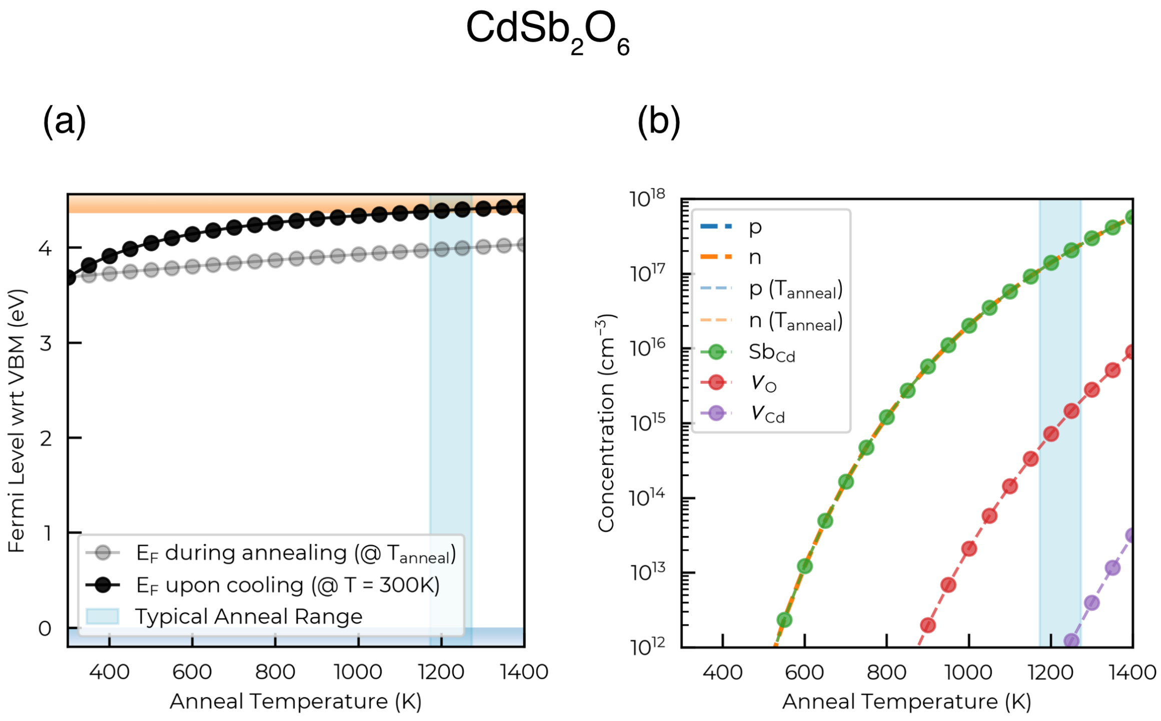

Figure 7: (a) Plot of the change in self-consistent Fermi level positions as a function of annealing temperature in undoped $CdSb_2O_6$. The grey line shows the E_F during annealing, while the black line represents the E_F after cooling to room temperature (300 K). The light blue shaded region shows the reported annealing temperature range.[1,6,7] (b) Room-temperature intrinsic carrier and defect concentrations versus annealing temperatures, where the defects with concentration below 10^{12} cm^{-3} are omitted from the legend.

Ga and F-doped $MgSb_2O_6$

Table 7: Formation energies per formula unit (ΔE_f) of all extrinsic competing phases included in Ga-doped $MgSb_2O_6$, with k-meshes used in calculations. Only the lowest energy polymorphs are included.

Formula	Space Group	k-mesh	ΔE_f (eV/fu)
Ga	Cmce	8×8×6	0.000
Ga_2O_3	C2/m	6×6×3	-10.040
$MgGa_2O_4$	Imma	4×4×4	-16.112
$GaSbO_4$	Cmmm	4×4×5	-10.333

Table 8: Chemical potential limits for Ga-doped $MgSb_2O_6$.

**Limit**	**Mg**	**Sb**	**O**	**Ga**	**Ga-Limiting Phase**
$Mg(SbO_3)_2$-$Mg_4Sb_2O_9$-O_2	-5.74	-5.57	0.00	-5.18	$Mg(GaO_2)_2$
Sb_2O_5-$Mg(SbO_3)_2$-O_2	-7.57	-4.66	0.00	-5.67	$GaSbO_4$
Sb_2O_5-$Mg(SbO_3)_2$-SbO_2	-6.83	-2.83	-0.73	-4.57	$GaSbO_4$
$Mg(SbO_2)_2$-$Mg(SbO_3)_2$-$Mg_4Sb_2O_9$	-3.73	-0.55	-2.01	-2.17	$Mg(GaO_2)_2$
Sb_2O_3-$Mg(SbO_3)_2$-SbO_2	-4.70	-0.70	-1.80	-2.44	$GaSbO_4$
Sb_2O_3-$Mg(SbO_2)_2$-$Mg(SbO_3)_2$	-4.07	-0.38	-2.01	-2.01	Ga_2O_3

Table 9: Formation energies per formula unit (ΔE_f) of all extrinsic competing phases included in F-doped $MgSb_2O_6$, with k-meshes used in calculations. Only the lowest energy polymorphs are included.

Formula	Space Group	k-mesh	ΔE_f (eV/fu)
F_2	P4/mmm	1×1×1	0.000
MgF_2	$P4_2$/mnm	6×6×8	-11.173
Sb_2F_7	$P2_1$/m	2×4×2	-21.687
SbF_3	Ama2	4×4×2	-9.661
SbF_4	$P2_1$/c	1×2×1	-11.988
SbF_5	$P2_1$/c	1×3×2	-13.841
$SbOF_3$	$P\bar{1}$	3×2×2	-10.188
Sb_4F_{15}	$Pca2_1$	2×1×1	-45.635
$Sb_{11}F_{43}$	$P2_1$/c	2×1×1	-129.493
Sb_7F_{29}	$P2_1$/c	1×2×1	-85.671
SbO_2F	Cc	4×4×3	-6.475

Table 10: Chemical potential limits for F-doped $MgSb_2O_6$.

**Limit**	**Mg**	**Sb**	**O**	**F**	**F-Limiting Phase**
$Mg(SbO_3)_2$-$Mg_4Sb_2O_9$-O_2	-5.74	-5.57	0.00	-2.71	MgF_2
Sb_2O_5-$Mg(SbO_3)_2$-O_2	-7.57	-4.66	0.00	-1.84	$SbOF_3$
Sb_2O_5-$Mg(SbO_3)_2$-SbO_2	-6.83	-2.83	-0.73	-2.29	Sb_2F_7
$Mg(SbO_2)_2$-$Mg(SbO_3)_2$-$Mg_4Sb_2O_9$	-3.73	-0.55	-2.01	-3.72	MgF_2
Sb_2O_3-$Mg(SbO_3)_2$-SbO_2	-4.70	-0.70	-1.80	-3.24	MgF_2
Sb_2O_3-$Mg(SbO_2)_2$-$Mg(SbO_3)_2$	-4.07	-0.38	-2.01	-3.55	MgF_2

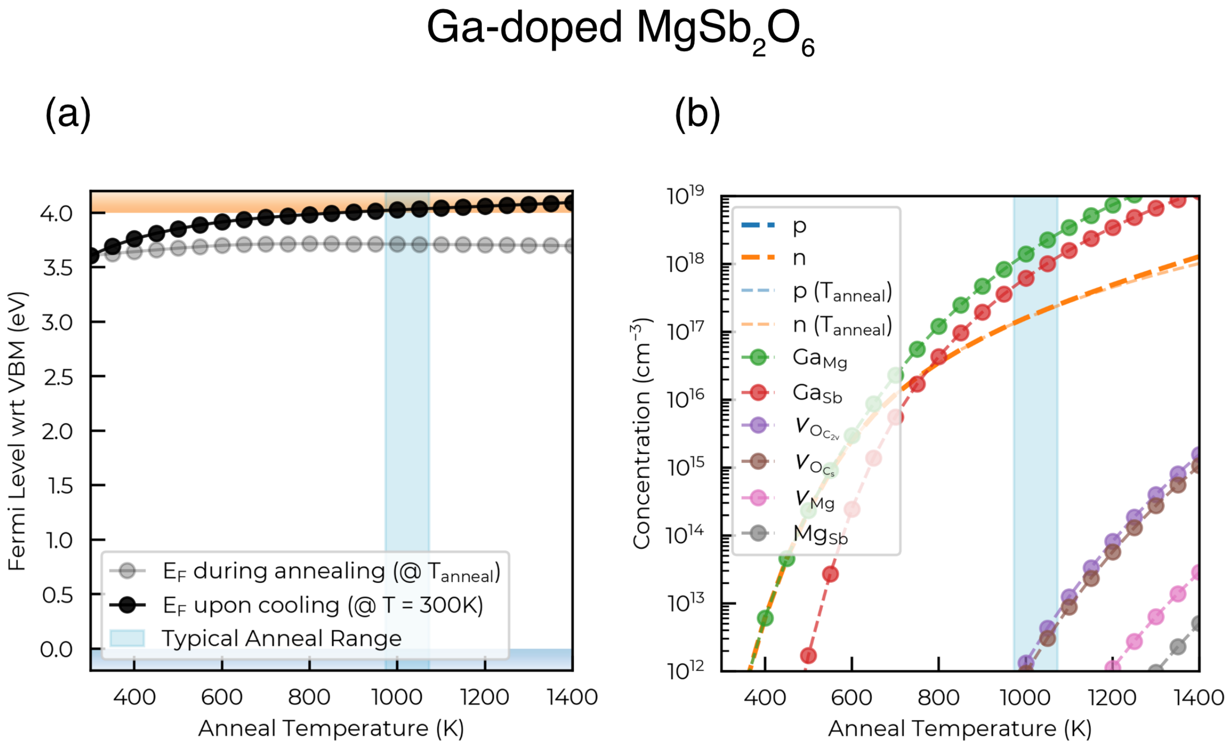

Figure 8: (a) Plot of the change in self-consistent Fermi level positions as a function of annealing temperature in Ga-doped $MgSb_2O_6$. The grey line shows the E_F during annealing, while the black line represents the E_F after cooling to room temperature (300 K). The light blue-shaded region indicates the reported annealing temperature range.[3–5] (b) Room-temperature carrier and defect concentrations versus annealing temperatures, where the defects with concentration below $1 \times 10^{12}\,cm^{-3}$ are omitted from the legend.

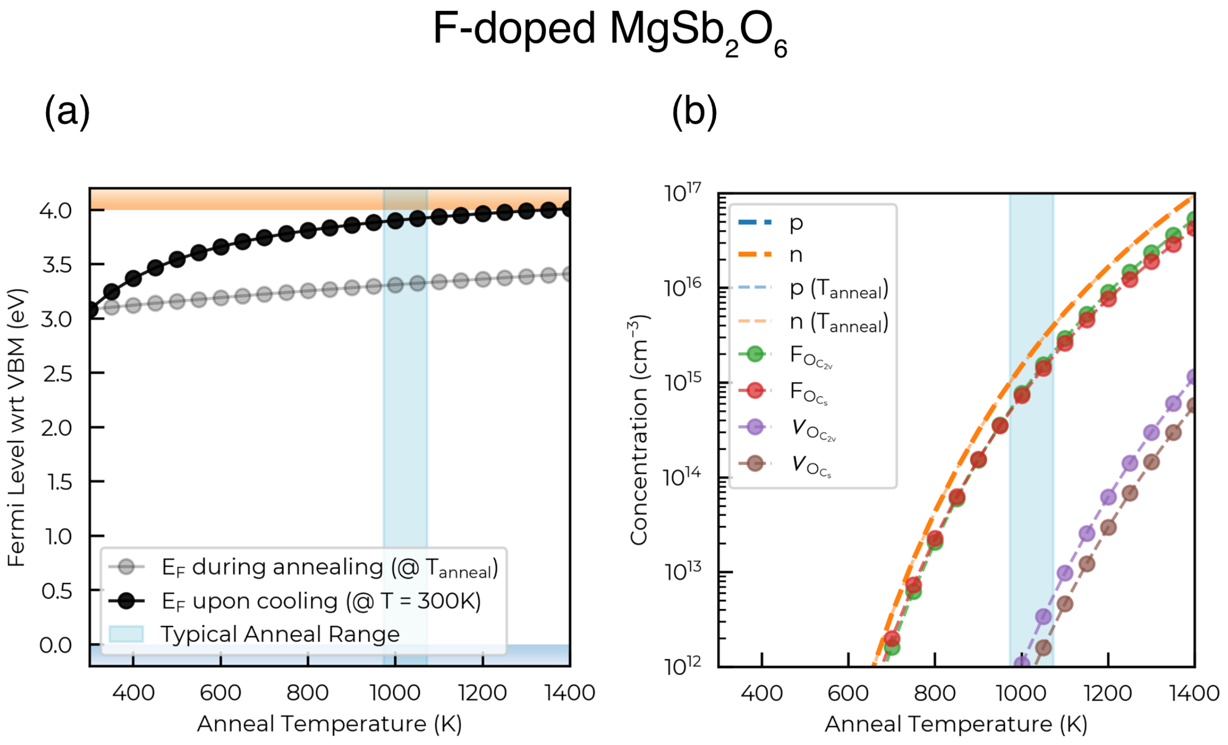

Figure 9: (a) Plot of the change in self-consistent Fermi level positions as a function of annealing temperature in F-doped $MgSb_2O_6$. The grey line shows the E_F during annealing, while the black line represents the E_F after cooling to room temperature (300 K). The light blue-shaded region indicates the reported annealing temperature range.[3–5] (b) Room-temperature carrier and defect concentrations versus annealing temperatures, where the defects with concentration below $1 \times 10^{12}\,cm^{-3}$ are omitted from the legend.

Y-doped $CdSb_2O_6$

Table 11: Formation energies per formula unit (ΔE_f) of all extrinsic competing phases included in Y-doped $CdSb_2O_6$. Only the lowest energy polymorphs are included.

Formula	Space Group	k-mesh	ΔE_f (eV/fu)
Y	R$\bar{3}$m	10×10×10	0.000
$Y_3Sb_5O_{12}$	I$\bar{4}$3m	3×3×3	-47.354
Y_3SbO_7	Cmcm	3×3×2	-35.242
$YSbO_4$	P$\bar{1}$	2×2×2	-15.497

Table 12: Chemical potential limits for Y-doped $CdSb_2O_6$.

**Limit**	**Cd**	**Sb**	**O**	**Y**	**Y-Limiting Phase**
$Cd(SbO_3)_2$-$Cd_2Sb_2O_7$-O_2	-2.67	-5.31	0.00	-10.19	$YSbO_4$
$Cd(SbO_3)_2$-Sb_2O_3-SbO_2	-1.08	-0.70	-1.80	-7.59	$YSbO_4$
$Cd(SbO_3)_2$-Sb_2O_3-$Cd_2Sb_2O_7$	-0.76	-0.54	-1.91	-7.32	$YSbO_4$
Sb_2O_5-$Cd(SbO_3)_2$-O_2	-3.95	-4.67	0.00	-10.83	$YSbO_4$
Sb_2O_5-$Cd(SbO_3)_2$-SbO_2	-3.21	-2.82	-0.74	-9.72	$YSbO_4$

Phonon band structures

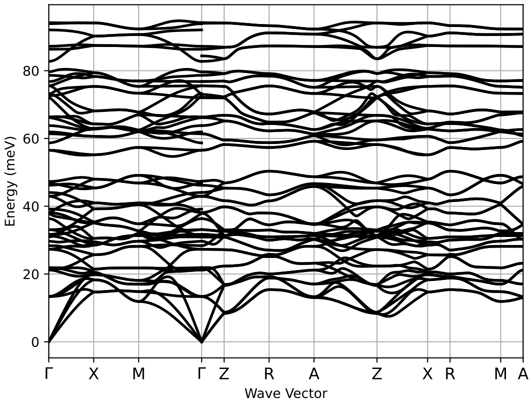

(a) $MgSb_2O_6$

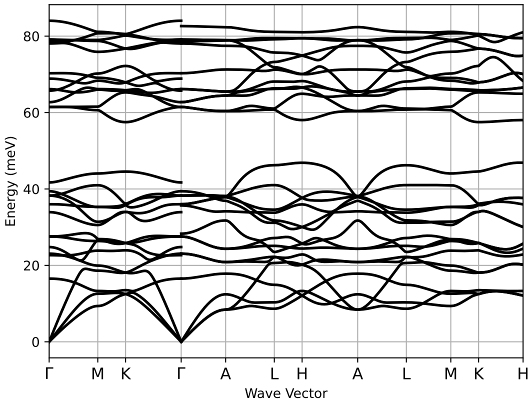

(b) $CaSb_2O_6$

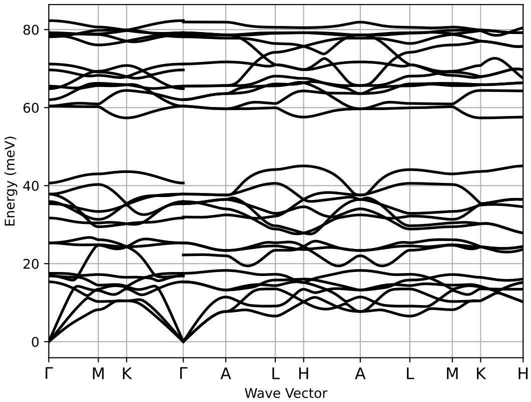

(c) $SrSb_2O_6$

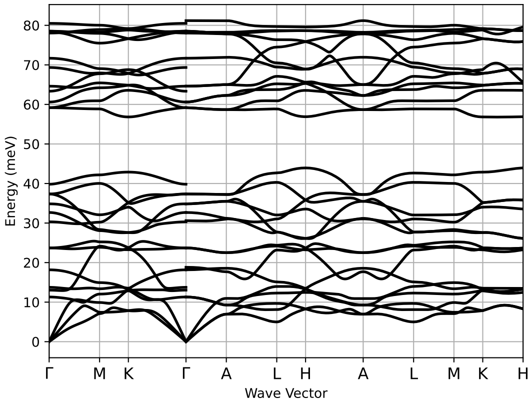

(d) $BaSb_2O_6$

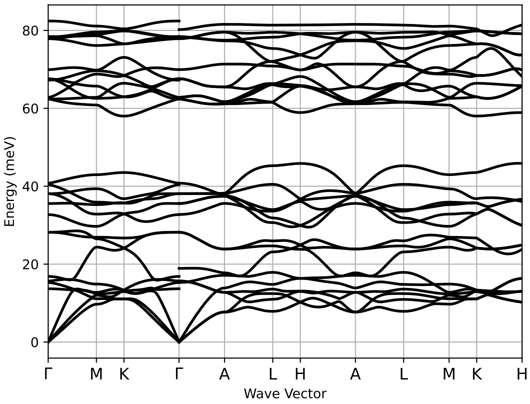

(e) $CdSb_2O_6$

Figure 10: Phonon band structures computed using DFPT and the PBEsol functional.

Experimental validation of $CdSb_2O_6$ and Y-doped $CdSb_2O_6$

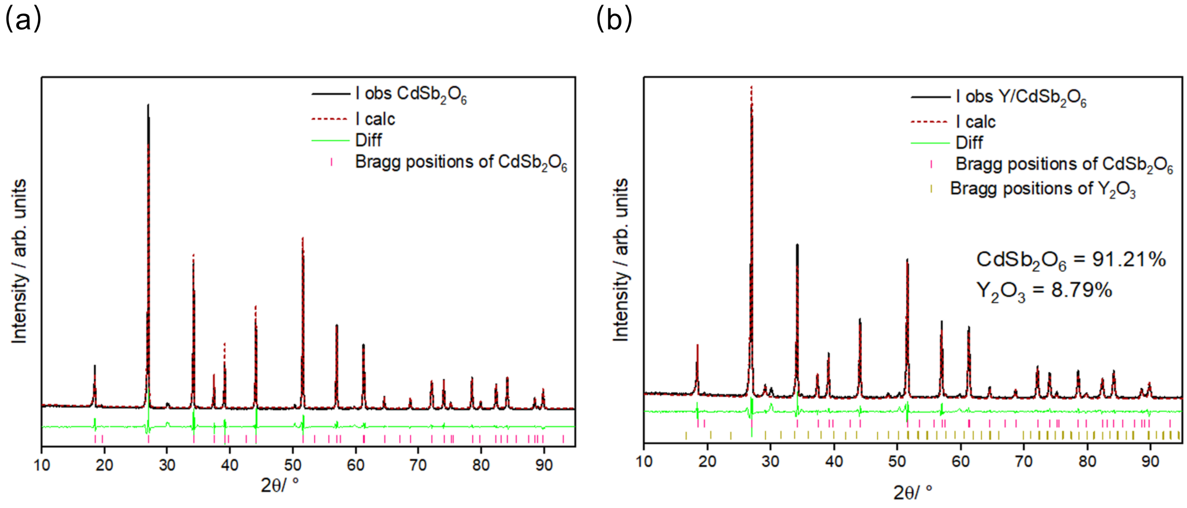

Figure 11: Rietveld refinement of Powder X-ray diffraction patterns for (a) pristine $CdSb_2O_6$ and (b) Y-doped $CdSb_2O_6$. Experimental (black), calculated (red), and difference (green) lines are shown, together with Bragg reflection positions. A minor Y_2O_3 secondary phase (∼8.79 %) is identified in the doped sample.

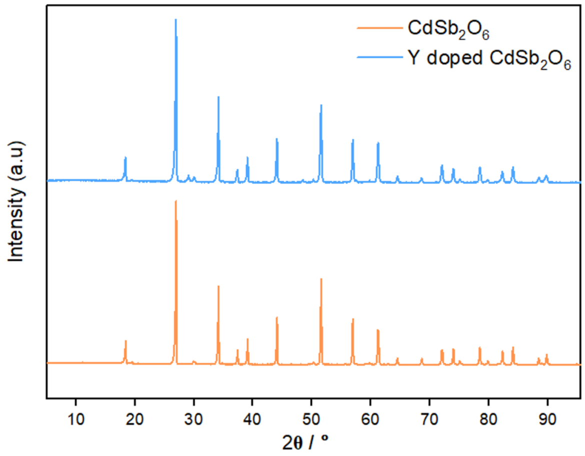

Figure 12: Powder X-ray diffraction patterns of pristine $CdSb_2O_6$ and Y-doped $CdSb_2O_6$. The diffraction peaks are consistent with the $CdSb_2O_6$ phase, with no significant peak shifts observed upon Y doping.

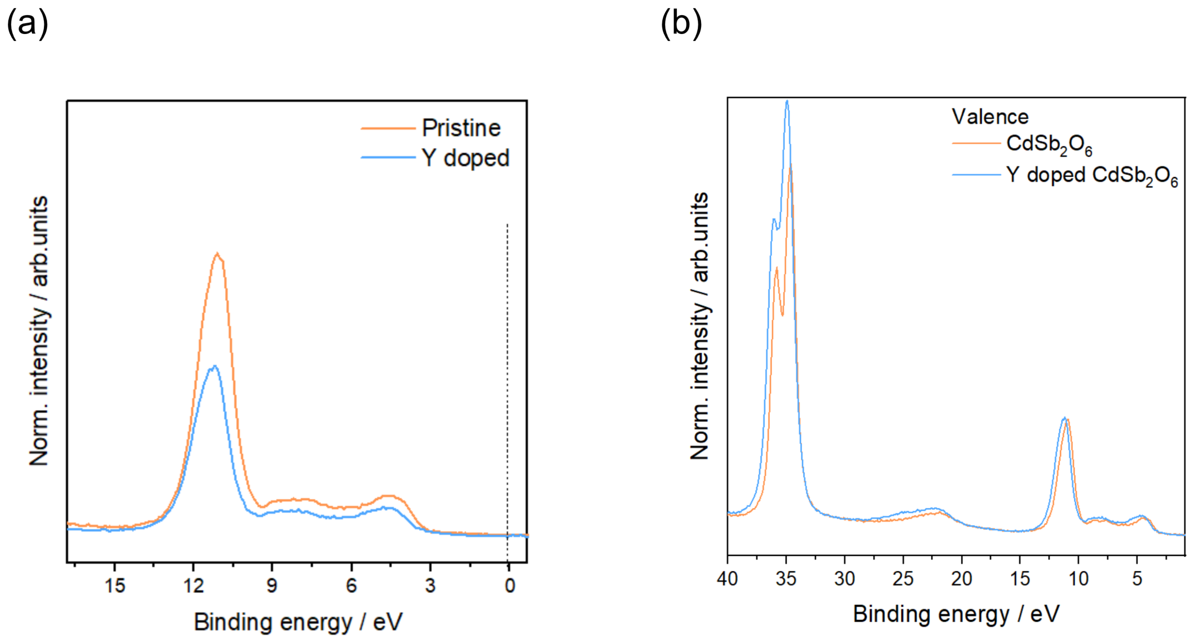

Figure 13: XPS spectra of $CdSb_2O_6$ and Y-doped $CdSb_2O_6$: (a) low binding energy region (b) valence band spectra. The valence band edge shows a shift upon Y-doping, consistent with increased carrier concentration and the Burstein-Moss shift.

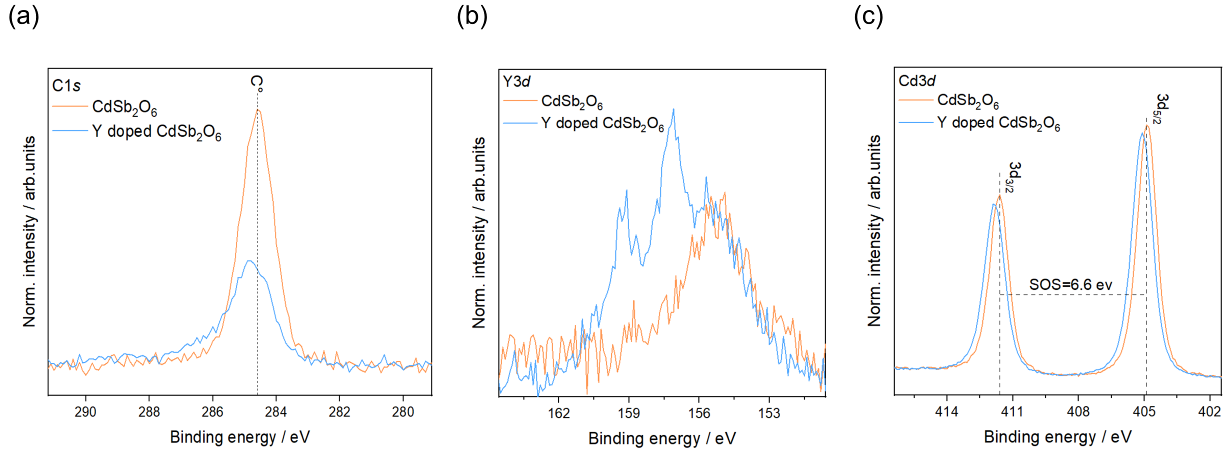

Figure 14: XPS spectra of $CdSb_2O_6$ and Y-doped $CdSb_2O_6$: (a) C 1*s* peak used for energy calibration; (b) Y 3*d* confirming incorporation of Y into the lattice; (c) Cd 3*d* core levels showing no significant shift, consistent with minimal perturbation of the host electronic structure upon Y-doping.

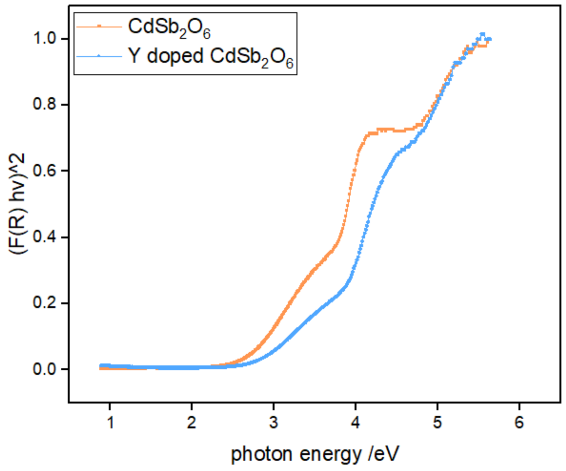

Figure 15: Overlay for the Tauc plots for pristine and Y-doped samples, highlighting the shift to higher energy upon Y-doping, consistent with a Burstein-Moss widening of the band gap.

References

(1) Mizoguchi, H.; Woodward, P. M. Electronic Structure Studies of Main Group Oxides Possessing Edge-Sharing Octahedra: Implications for the Design of Transparent Conducting Oxides. *Chemistry of Materials* **2004**, *16*, 5233–5248.

(2) DeBoer, B. G.; Young, R. A.; Sakthivel, A. X-ray Rietveld structure refinement of Ca, Sr and Ba meta-antimonates. *Acta Crystallographica Section C Crystal Structure Communications* **1994**, *50*, 476–482.

(3) Guillén-Bonilla, H.; Flores-Martínez, M.; Rodríguez-Betancourtt, V.-M.; Guillen-Bonilla, A.; Reyes-Gómez, J.; Gildo-Ortiz, L.; De La Luz Olvera Amador, M.; Santoyo-Salazar, J. A Novel Gas Sensor Based on $ZnSb_2O_6$ Nanorods to Indicate Variations in Carbon Monoxide and Propane Concentrations. *Sensors* **2016**, *16*, 177.

(4) Juárez-Amador, L. I.; Guillén-Bonilla, H.; Guillén-Bonilla, A.; Guillén-Bonilla, J. T.; Morales-Bautista, J.; Casillas-Zamora, A.; Rodríguez-Betancourtt, V.-M.; Olvera-Amador, M. D. L. L. Photocatalytic and sensing properties in propane atmospheres

of $MgSb_2O_6$ nanoparticles synthesized by a chemical method. *Journal of Materials Science: Materials in Electronics* **2024**, *35*, 1857.

(5) Guillén-Bonilla, H.; Guillén-Bonilla, J. T.; Rodríguez-Betancourtt, V.-M.; Ramírez-Ortega, J. A.; Morán Lázaro, J. P.; Guillén-Bonilla, A. Synthesis and Sensing Response of Magnesium Antimoniate Oxide ($MgSb_2O_6$) in the Presence of Propane Atmospheres at Different Operating Voltages. *Sensors* **2024**, *24*, 2147.

(6) Dutta, D. P.; Ballal, A.; Singh, A.; Fulekar, M. H.; Tyagi, A. K. Multifunctionality of rare earth doped nano $ZnSb_2O_6$, $CdSb_2O_6$ and $BaSb_2O_6$: photocatalytic properties and white light emission. *Dalton Transactions* **2013**, *42*, 16887.

(7) Majzlan, J.; Jia, X.; Lilova, K.; Subramani, T.; Navrotsky, A.; Dachs, E.; Benisek, A. Thermodynamic stability of selected ASb_2O_6 and ASb_2O_7 phases (A = Ca, Ba, Cd, Sr, Zn). *Solid State Sciences* **2024**, *154*, 107615.
